# A ubiquitin-like domain controls protein kinase D dimerization and activation by trans-autophosphorylation

**DOI:** 10.1074/jbc.RA119.008713

**Published:** 2019-08-12

**Authors:** Daniel J. Elsner, Katharina M. Siess, Thomas Gossenreiter, Markus Hartl, Thomas A. Leonard

**Affiliations:** ‡ Department of Structural and Computational Biology, Max Perutz Labs, Campus Vienna Biocenter 5, 1030 Vienna, Austria; § Mass Spectrometry Facility, Max Perutz Labs, Dr. Bohr-Gasse 3, 1030 Vienna, Austria; ¶ Department of Biochemistry and Cell Biology, Max Perutz Labs, University of Vienna, Vienna Biocenter, Dr. Bohr-Gasse 9, 1030 Vienna, Austria; ‖ Department of Medical Biochemistry, Medical University of Vienna, 1090 Vienna, Austria

**Keywords:** protein kinase D (PKD), autophosphorylation, signal transduction, diacylglycerol, dimerization, crystal structure, ubiquitin-like domain, second messenger, structural biology

## Abstract

Protein kinase D (PKD) is an essential Ser/Thr kinase in animals and controls a variety of diverse cellular functions, including vesicle trafficking and mitogenesis. PKD is activated by recruitment to membranes containing the lipid second messenger diacylglycerol (DAG) and subsequent phosphorylation of its activation loop. Here, we report the crystal structure of the PKD N terminus at 2.2 Å resolution containing a previously unannotated ubiquitin-like domain (ULD), which serves as a dimerization domain. A single point mutation in the dimerization interface of the ULD not only abrogated dimerization in cells but also prevented PKD activation loop phosphorylation upon DAG production. We further show that the kinase domain of PKD dimerizes in a concentration-dependent manner and autophosphorylates on a single residue in its activation loop. We also provide evidence that PKD is expressed at concentrations 2 orders of magnitude below the ULD dissociation constant in mammalian cells. We therefore propose a new model for PKD activation in which the production of DAG leads to the local accumulation of PKD at the membrane, which drives ULD-mediated dimerization and subsequent trans-autophosphorylation of the kinase domain.

## Introduction

Protein kinase D (PKD)^3^ is at the heart of a variety of cellular functions and essential for mammalian development (1). PKD is involved in such fundamental processes as vesicular trafficking at the *trans*-Golgi network (TGN) (2) and mitogenesis (3) but has also been proposed to play a role in more specialized processes, such as insulin secretion in pancreatic β-cells (4) and inflammasome activation in macrophages (5). Mutations in PKD have been associated with various cancers (6, 7), and its role in invasive breast cancer has been extensively investigated (reviewed in Ref. 8). Furthermore, dysregulation of PKD has been linked to several pathologies, such as cardiac hypertrophy (1) and oncogene-induced senescence (9). Given that PKD is involved in so many different processes and pathologies, the question of how PKD activity is governed in the cell is of outstanding interest. However, relatively little is understood about the regulatory mechanisms involved, in part due to the absence of structural information for this important enzyme.

**Figure 1.**
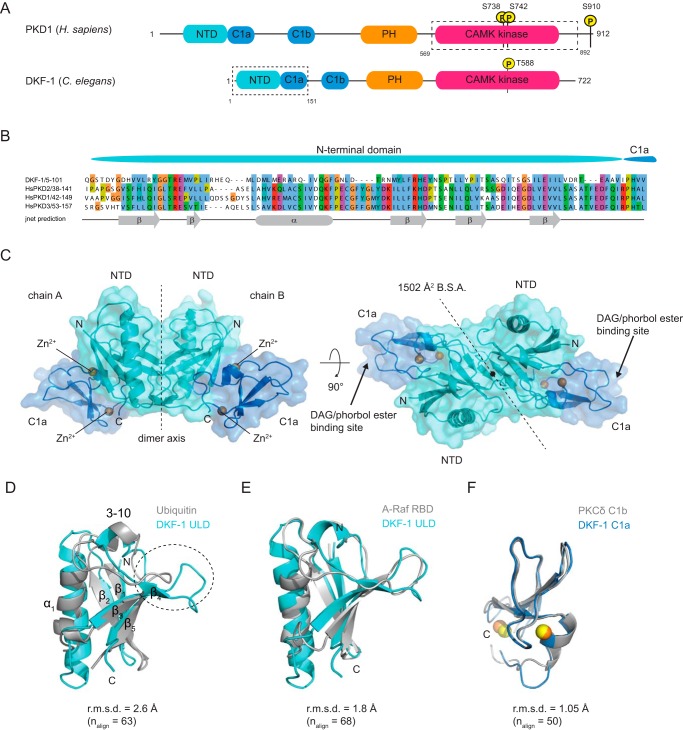
**The PKD N terminus contains a ubiquitin-like domain.** *A*, canonical domain architecture of PKDs illustrated by *Homo sapiens* PKD1 and *C. elegans* DKF-1; N-terminal domain (NTD), C1a, C1b, PH, and kinase domain belonging to the CAMK family. The putative NTD was identified by bioinformatics analysis. The *dashed boxes* indicate the construct used for crystallization (DKF-1^1–151^) and autophosphorylation experiments (PKD1^569–892^). Domains and linkers are drawn to scale. *B*, alignment of the N-terminal domain of *C. elegans* DKF-1 with the three human PKD isoforms. Secondary structure prediction of the PKD N terminus reveals the presence of a putative domain composed of potential β-sheets (β) and one α-helix (α). *C*, representation of the crystal structure of the dimeric DKF-1^1–151^ with NTD in *cyan*, C1a in *blue*, and Zn^2+^ ions in *orange. Arrows* indicate the DAG/phorbol ester binding cleft (by homology). *B.S.A.*, buried surface area. *D*, superposition of the NTD (*DKF-1 ULD*) with ubiquitin (PDB entry 1UBQ). The secondary structure elements are annotated (see also Fig. S1*B*). *E*, superposition of the NTD (*DKF-1 ULD*) with the Ras-binding domain (*RBD*) of A-Raf (PDB entry 1WXM). *F*, superposition of the C1a domain (DKF-1 C1a) with the C1b domain of PKCδ (PKCδ C1b) (PDB entry 1PTQ).

Protein kinase D is a serine-threonine kinase comprising two N-terminal C1 domains, a PH domain and a C-terminal kinase domain belonging to the Ca^2+^/calmodulin-dependent kinase (CAMK) family (Fig. 1*A*). PKD signaling is tightly controlled by the lipid second messenger diacylglycerol (DAG), which in cells is produced by phospholipase C (PLC) downstream of either GPCR (10) or receptor tyrosine kinase signaling (11). DAG recruits PKD via its two C1 domains to specific membranes in the cell, such as the plasma membrane (12, 13) and the TGN (14), where PKD is reportedly active (15).

In addition to recruiting PKD to membranes, DAG also activates PKD directly. Immunoprecipitated PKD exhibits a higher catalytic activity in the presence of DAG or DAG-mimicking phorbol esters *in vitro* (16), whereas removal of the regulatory C1 domains results in a kinase insensitive to DAG/phorbol ester stimulation, but with increased basal activity (17). Other studies have reported that deletion of the PH domain also increases PKD catalytic activity (18, 19). It has therefore been proposed that the C1 and PH domains maintain PKD in an inactive, autoinhibited state that is activated by binding of the C1 domains to membranes containing DAG.

A second requirement for PKD activation is the phosphorylation of two serine residues (Ser^738^ and Ser^742^ in human PKD1) in the activation loop of the kinase domain (20). Although the addition of phorbol esters has been shown to be sufficient for PKD activation (16) and activation loop autophosphorylation *in vitro* (21), previous studies have implicated the novel PKCs in PKD phosphorylation (22, 23). More recent studies have indicated that PKD autophosphorylation occurs *in vivo* on at least one of the two sites (Ser^742^ in human PKD1) in the activation loop in a PKC-independent manner (3, 24), raising questions about the necessity of upstream kinases for PKD activation.

A functional requirement for PKD dimerization *in vivo* has been reported previously (25, 26). These papers have implicated the N terminus of PKD in mediating dimerization, but the structural basis of dimerization and its role in PKD activation are currently unknown. Here we report the crystal structure of the N terminus of the *Caenorhabditis elegans* PKD homolog DKF-1. We find that the PKD N terminus includes a previously unannotated ubiquitin-like domain (ULD) and demonstrate that this domain is a novel dimerization domain. Mutation of the dimerization interface abrogates dimerization of the ULD *in vitro* and full-length PKD in cells. Impairment of ULD-mediated dimerization prevents PKD activation *in vivo*. Consistent with this, we find that the isolated PKD kinase domain dimerizes in a concentration-dependent manner and autophosphorylates on Ser^742^ *in vitro*. We therefore propose a new model of PKD autoactivation in which DAG drives the local concentration of PKD followed by ULD-mediated dimerization and kinase domain trans-autophosphorylation.

## Results

### The N terminus of PKD comprises a ubiquitin-like domain

Bioinformatic analysis revealed that the PKD N terminus is composed of a stretch of conserved amino acids. Secondary structure prediction (27) indicated this region to be ordered and to contain secondary structure elements that suggested a previously unannotated N-terminal domain (Fig. 1, *A* and *B*). A sequence alignment of distantly related PKD isoforms of the metazoan kingdom (Fig. S1) showed that this domain is immediately followed by the C1a domain in all species, unlike the other regulatory domains of PKD, which are separated by linkers of variable length (Fig. 1*A*). This suggested that the two most N-terminal domains might have evolved as a single functional unit.

To determine the structure of this module, a construct comprising the N-terminal and C1a domain of the *C. elegans* PKD homolog DKF-1 was purified and crystallized. The mass of the purified protein was confirmed by MS (Fig. S2*A*). Crystals diffracted to 2.2 Å, and the structure could be solved by experimental single anomalous dispersion phasing using the anomalous scattering of the Zn^2+^ ions in the C1a domain (Table S1). The crystal structure reveals a dimeric assembly of the protein, in which the dimer interface sits on a crystallographic axis (Fig. 1*C*). Dimerization is mediated by the N-terminal domain, which adopts a ubiquitin-like fold (Fig. 1*D* and Fig. S1*B*). Henceforth, we refer to the N-terminal domain as a ULD. Using PDBeFold, the ULD structure was compared with other ubiquitin-like domains in the Protein Data Bank (PDB), which showed that the DKF-1 ULD has highest structural similarity to the Ras-binding domain (RBD) of the serine-threonine kinase A-Raf (Fig. 1*E*) with an overall root mean square deviation (r.m.s.d.) of 1.8 Å over 63 C_α_ atoms. The C1 domain has highest structural homology to the DAG/phorbol ester-binding C1b domain of PKCδ with an r.m.s.d. of 1.05 Å over 50 C_α_ atoms (PDB entry 1PTQ) (28) (Fig. 1*F*). Intriguingly, the RBD of Raf is also closely followed by a membrane-binding C1 domain, although there is no obvious functional similarity.

### The ULD dimerizes via a conserved hydrophobic patch in a concentration-dependent manner

**Figure 2.**
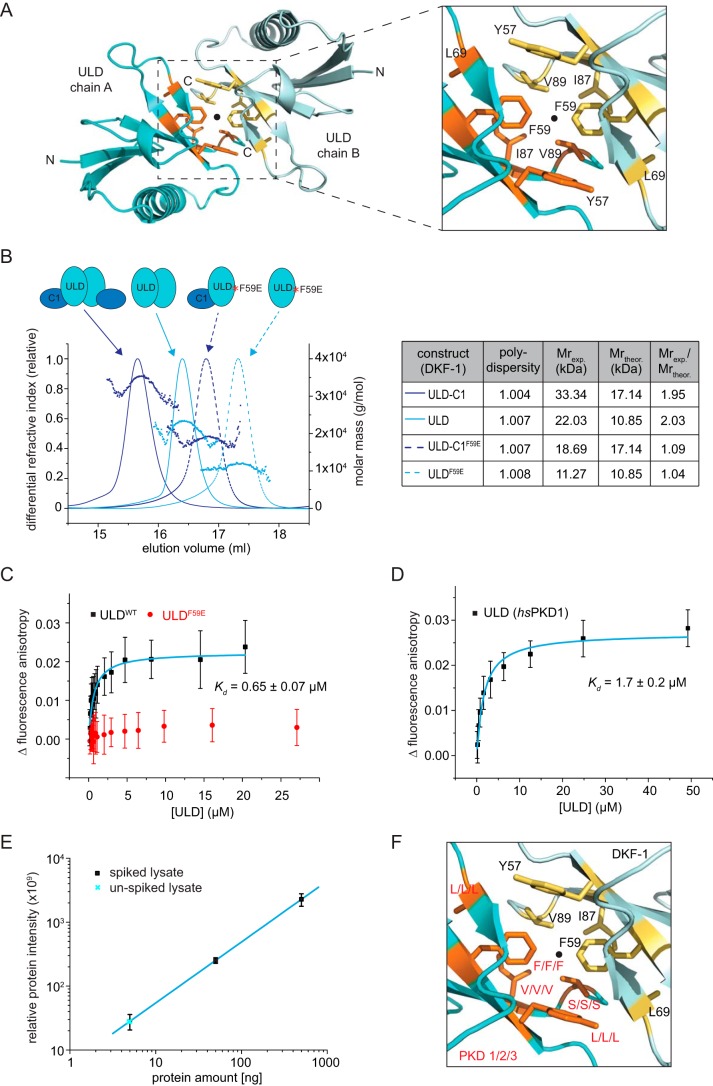
**Concentration-dependent dimerization of the ULD via a hydrophobic patch.** *A*, the ULD dimerization interface buries 1502 Å^2^ of surface area and is predominantly hydrophobic in character. *B*, static light scattering (SEC-MALS) of the ULD–C1a construct (*purple*, *solid line*) and the ULD alone (*cyan*, *solid line*) and the same proteins carrying a phenylalanine-to-glutamate point mutation (F59E) in the dimerization interface (*dashed lines*). The *table* summarizes the polydispersity (*Mn*/*Mw*) as well as the experimentally determined molecular weight (*M*_exp_), the theoretical monomeric molecular weight (*M*_theor_), and the oligomeric state (*M*_exp_/*M*_theor_) of each protein. *C*, titration of DKF-1 ULD (ULD^WT^) or ULD^F59E^ into a fixed amount of Atto488-labeled ULD^R42C^. The change in fluorescence anisotropy is plotted against the concentration of ULD^WT^/ULD^F59E^ ([ULD]). Data points represent the mean and S.D. (*error bars*) of 29–37 technical replicates. For the ULD, data points were fitted with a single-site binding model (*cyan line*) to derive the equilibrium dissociation constant (*K_d_*) of ULD dimerization. *D*, equilibrium dissociation constant (*K_d_*) of human PKD1^ULD^ determined by fluorescence anisotropy. The fluorescence anisotropy of 40 nm Atto488-ULD increases when unlabeled ULD is added to it. Data points are the mean and S.D. of 26–63 technical replicates and were fitted with a single binding model to derive the *K_d_* of dimerization. *E*, summed, globally normalized intensities of five proteotypic PKD1 peptides targeted by parallel reaction monitoring MS for different amounts (5, 50, 500 ng) of spiked-in recombinant PKD1 protein (*black squares*) or unspiked cell lysates (*cyan crosses*). From each spiked sample, the signal of the unspiked sample was subtracted. S.D. is from three biological replicates. The linear regression of the spiked samples was used to calculate the amount of endogenous PKD1 in unspiked cells. *F*, *close-up* of the dimerization interface as in Fig. 2*A*. All hydrophobic residues in the interface are *highlighted* as *sticks*, one protomer in *yellow* and the other one in *orange*. Not all of the residues are identical between DKF-1 (*black*) and the three human isoforms (PKD1/2/3, *red label* at corresponding positions in the DKF-1 structure). However, all residues in the interface are identical for all three human PKD isoforms.

The dimerization interface on the ULD is mostly composed of hydrophobic residues (Fig. 2*A*). These residues form a surface that overlaps with the hydrophobic patch in ubiquitin, which plays an essential role in ubiquitin recognition of effector proteins (29). Use of this surface for dimerization, however, has not previously been reported. Using the structure of the isolated ULD dimer (residues 12–98) and the PDBePISA server, an interface area of 751 Å^2^ and a solvation free energy (Δ*G*) of −5.4 kcal/mol were calculated for the dimerization interface. Many of the residues in the ULD dimerization interface are well-conserved among distantly related PKD homologs, with a central phenylalanine being invariant from nematodes and arthropods to chordates and mammals (Fig. S1). Static light scattering (SEC-MALS) showed that the ULD and the ULD–C1a are monodisperse and dimeric in solution (Fig. 2*B*). To validate the dimerization interface observed in the crystal structure, we mutated the central hydrophobic phenylalanine to a negatively charged glutamate (F59E), the rationale being that two negatively charged residues in the interface should repel each other. Indeed, the F59E mutation converted the ULD and ULD–C1a into exclusive monomers (Fig. 2*B*). Additionally, we also confirmed that the ULD of human PKD1 and PKD3 were dimeric by SEC-MALS (Fig. S3*A*). This observation as well as the conservation of the ULD and in particular the conservation of the dimerization interface suggests that dimerization of the N-terminal ULD is common to all PKD isoforms.

Serial SEC of the crystallized *C. elegans* ULD–C1a indicated a shift in the retention time toward a monomeric species as a function of protein dilution (Fig. S3*B*), suggesting that ULD dimerization is concentration-dependent. Notably, the ULD–C1a protein eluted as a single symmetric peak at all concentrations, suggesting that the kinetics of association and dissociation must be fast and cannot be resolved by SEC. We next determined the affinity of ULD dimerization by fluorescence anisotropy (Fig. 2*C*). Although we could determine the equilibrium dissociation constant (*K_d_*) for the WT DKF-1 ULD to be 0.65 ± 0.07 μm, we were unable to detect any change in fluorescence anisotropy for the F59E mutant. This confirms that this point mutation abolishes the ability of the ULD to dimerize. We also determined the equilibrium dissociation constant for the ULD dimer of human PKD1, the most studied isoform of this kinase family, and derived a *K_d_* of 1.7 ± 0.2 μm (Fig. 2*D*).

The high-nanomolar to low-micromolar affinity for the ULD raised the question of whether ULD dimerization could be constitutive in the cell. We therefore sought to estimate the concentration of endogenous PKD1 in HEK239T cells by parallel reaction monitoring (PRM) MS. To this end, we generated recombinant ULD–C1a and PH-CAT proteins of PKD1 and used these proteins to spike HEK293T cell lysates of known cell number. After tryptic digestion, we quantified the individual PKD1-specific peptides by targeted MS (see supporting Experimental procedures for details). We determined the amount of endogenous PKD1 to be 4.9 ± 0.4 ng in the HEK293T lysates (Fig. 2*E*), which corresponds to an overall cellular concentration of 31.9 ± 0.5 nm, or an exclusively cytosolic concentration of 69.8 ± 1.1 nm, respectively. We also investigated the levels of endogenous PKD1 in INS-1 cells. This is a cell line derived from pancreatic β-cells of *R. norvegicus*, in which PKD has a well-characterized signaling function (4, 30, 31). Although our PRM approach for these cells is limited because only three suitable peptides are identical between the spiked human PKD1 and endogenous rat PKD1 protein, we were able to quantify two of these peptides (Fig. S3*C*). The amount of quantified peptide (1.76–5.29 ng) corresponds to a cellular concentration of 6.2–18.5 nm, suggesting that the overall PKD1 concentration in INS-1 cells is in the low- to mid-nanomolar range. Taken together, we found that the cellular concentrations of PKD1 in HEK293T and INS-1 cells are around 2 orders of magnitude lower than the *K_d_* of ULD dimerization (1.7 ± 0.2 μm), which makes ULD dimerization unlikely to be constitutive in these cells.

We also noticed that the symmetric dimerization interface is not only highly conserved between different orthologues of PKD but is in fact identical between the three human PKD isoforms (Fig. S1 and Fig. 2*F*). We therefore hypothesized that the ULD must also function as a heterodimerization domain between the PKD paralogs. We addressed this possibility by co-expressing the three human homologs of the ULD–C1a protein and assessed the ability of these proteins to heterodimerize in pulldown assays. GST-tagged human PKD3 was able to pull down the co-expressed and His-tagged PKD1 and PKD2 isoforms from bacterial cell lysates (Fig. S3*D*), supporting our assumption that the ULD functions both as a homo- and heterodimerization domain for PKD.

### ULD dimerization restricts the orientation of the membrane-binding sites of the C1 domain

**Figure 3.**
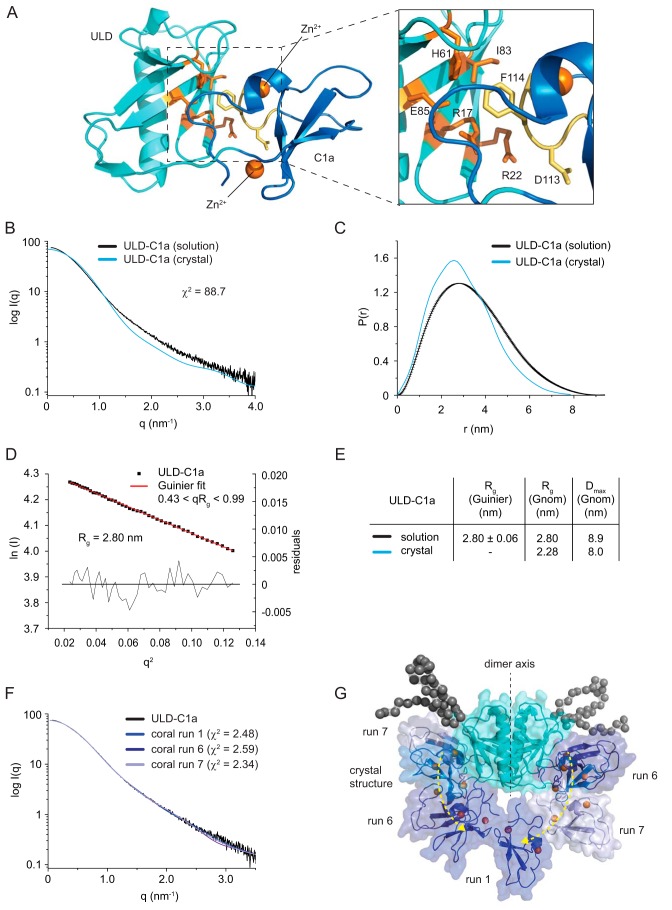
**The ULD dimer restricts the orientation of the membrane-binding sites of the C1 domain.** *A*, The orientation of the C1 domain with respect to the ULD is mediated by a patch of conserved residues on the ULD (*orange*) and C1a (*yellow*). *B*, small-angle X-ray scattering curve of the crystallized protein (DKF-1^1–151^) in solution (*black line*) compared with the theoretical scattering curve calculated from the crystal structure (*blue line*) using CRYSOL (32). The high χ^2^ value indicates that the crystal structure does not match the solution scattering. *C*, pair distribution function of DKF-1^1–151^ derived from the SAXS data (*black line*) and the crystal structure (*blue line*). *D*, Guinier plot of the solution scattering data. The interval of 0.43 < *qR_g_* < 0.99 was used to determine the radius of gyration (*R_g_*) by a linear regression of the data. Residuals of the fit are shown *below. E*, comparison of the *R_g_* and the maximum dimension (*D*_max_) of the ULD–C1a protein derived from the solution-scattering data (*solution*) and calculated from the crystal structure (*crystal*). The radius of gyration was derived either from the Guinier plot or from the pair distribution function. *F*, rigid-body modeling of the ULD–C1a using CORAL (32). Shown is the *scattering curve* of the ULD–C1a in solution (*black line*) and theoretical scattering curves of models that best fit the experimental scattering. The χ^2^ values represent the goodness of fit of the models to the experimental data. *G*, superposition of the crystal structure and the structures obtained by rigid-body modeling with the lowest χ^2^ values (see *F*). *Black spheres* represent dummy spheres for N-terminal residues not visible in the crystal structure.

Aside from ULD dimerization, the structure also revealed an intramolecular interaction between the ULD and its corresponding C1a domain (Fig. 3*A*). This interface, in combination with the dimerization interface, gives rise to a unique arrangement of the C1 domains, with their respective DAG binding sites pointing in almost exactly opposite directions (Fig. 1*C*) and contains three highly conserved residues in the ULD, Arg^17^, Arg^22^, and Glu^85^, that form a contiguous surface on the domain outside of the dimer interface. Arg^17^ appears to stabilize the ULD–C1a interaction by formation of a hydrogen-bond network with the side chain of Glu^85^ and the backbone carbonyl oxygens of Arg^145^ and Phe^114^ in the C1a domain (Fig. S4*A*). However, Arg^17^ is actually a glutamine in other PKD orthologs, and Arg^22^, which is invariant (Fig. S1), is solvent-exposed (Fig. S4*B*). The high degree of conservation in the interface between the ULD and the C1a domain (Fig. 3*A* and Fig. S1) suggests that these residues may be functionally important, but it does not exclude the possibility that the ULD–C1a interaction observed in the crystal structure could be an artifact of crystal packing. We therefore investigated the domain arrangement in solution by small-angle X-ray scattering (SAXS). The experimentally determined scattering curve of the ULD–C1a deviated substantially from the calculated scattering curve of the crystal structure (Fig. 3*B*). Similarly, the pair distribution function, radius of gyration, and maximum dimension of the ULD–C1a differed significantly between the solution-scattering data and the expected values (Fig. 3, *C–E*), suggesting that the conformation of the ULD–C1 protein in solution is not identical to the one observed in the crystal structure. We then performed rigid-body modeling using CORAL (32). Given that the SAXS data were measured under experimental conditions in which the ULD–C1a protein is dimeric (Fig. 2*B*), we fixed the two ULDs as a single rigid body and allowed the C1 domains to sample conformational space limited by the length of the ULD–C1a interdomain linker. Using this approach, several conformations could be modeled whose calculated scattering curves fit well to the experimental scattering data (Fig. 3*F*). Overall, these structures exhibited displacement of one or both C1 domains from the ULD (Fig. 3*G*), suggesting an equilibrium of conformational states in solution.

**Figure 4.**
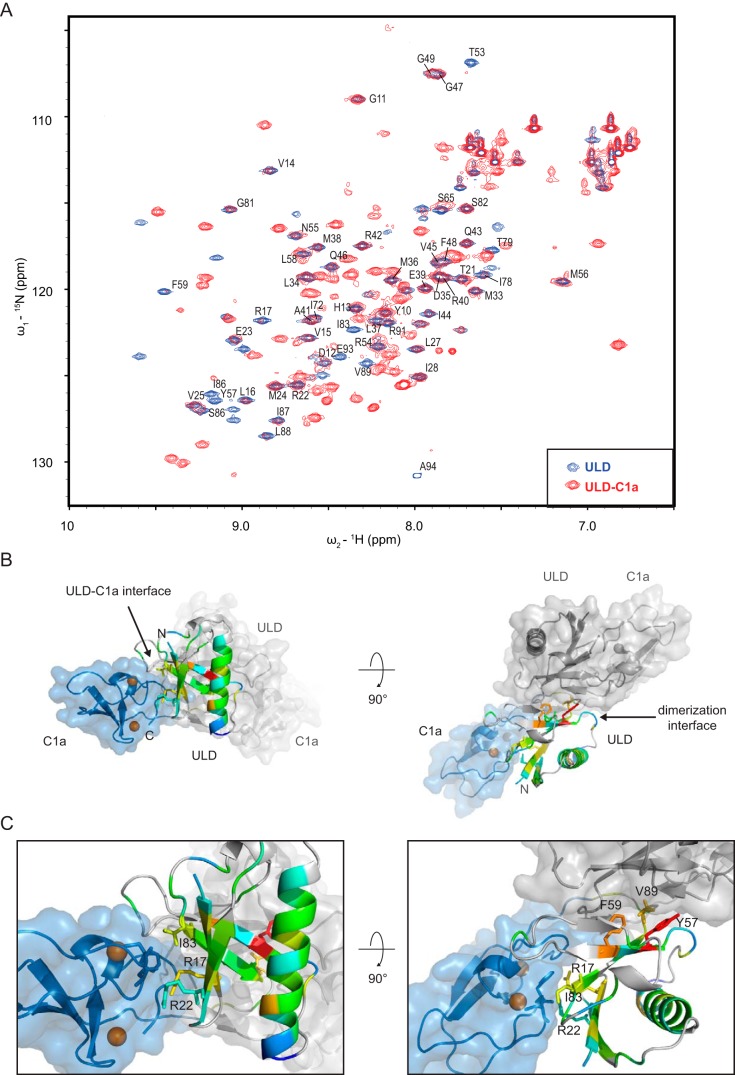
**The ULD and C1a domain interact reversibly in solution.** *A*, overlay of ^15^N and ^1^H resonances of two HSQC experiments on DKF-1^1–94^ (*ULD*, *blue peaks*) and DKF-1^1–151^ (*ULD–C1a*, *red peaks*). *B*, changes in peak intensities of the amide backbone ^15^N and ^1^H cross-resonances as seen in Fig. 4*A* were mapped onto the ULD of the crystal structure. The *color code* is from *blue* (no decrease in intensity) to *red* (highest change in intensity). Peaks that could not be assigned are represented in *gray. C*, *close-up* of *B*. Some of the residues in the ULD C1a interface (Arg^17^, Arg^22^, and Ile^83^), but also in the dimerization interface (Tyr^57^, Phe^59^, and Val^89^) display altered intensity.

The interaction of the two domains was further investigated by solution NMR using the ULD domain in isolation and the ULD–C1a protein for which the crystal structure was determined. In the case of the ULD, the two-dimensional ^15^N-^1^H HSQC spectrum showed well-defined and well-resolved cross-peaks for the amide backbone resonances, as expected for a small, compact domain (Fig. 4*A*). We then sequentially assigned the backbone resonances of the ULD spectrum using standard 3D triple-resonance techniques. In contrast, when comparing the HSQC spectrum of the ULD with that obtained for the ULD–C1a protein, most of the resonances that showed well-defined cross-peaks in the ULD spectrum displayed substantial peak broadening and subsequent reduction in intensity in the ULD–C1a protein, which suggests that the ULD and C1a domains are not independent entities and interact with each other in solution. This change in peak shape and intensities can be explained by the altered relaxation properties of the affected amino acids in the two-domain construct and is the result of conformational dynamics at the interface between the two domains. The data are consistent with a reversible binding/dissociation event of the C1a to the ULD occurring in the high-microsecond to low-millisecond time scale. However, beyond the resonances of the amino acids located in the interface between the ULD and C1a domain, we also found residues outside the ULD–C1a interface whose resonances were affected by the presence of the C1a domain (Fig. 4, *B* and *C*). This indicates that the C1a domain elicits global changes in the chemical environment of the ULD, extending also to the dimerization interface.

Taken together, our data suggest that the ULD and C1 domain interact reversibly in solution, existing in a steady equilibrium of different conformations, with the crystal structure representing presumably the most compact state. Our findings imply that the ULD restricts the orientation of the C1 domains, which could be important for target membrane recognition.

### ULD dimerization is required for PKD activation in cells

**Figure 5.**
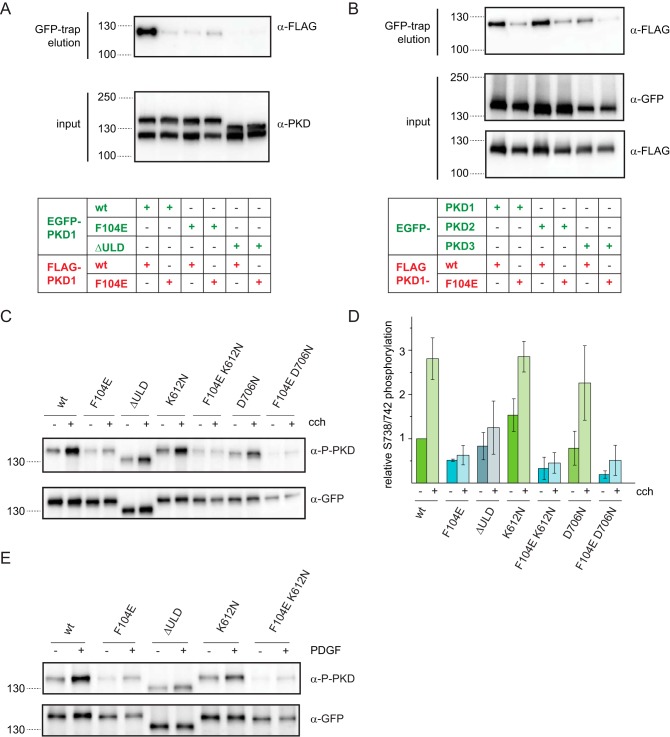
**ULD dimerization is required for PKD activation in cells.** *A*, EGFP- and FLAG-tagged PKD1 constructs were co-expressed in HEK293T cells and subjected to co-immunoprecipitation using GFP-trap. Expression levels of transgenes and co-immunoprecipitated proteins were detected by Western blotting using a PKD-specific antibody (α-PKD) and FLAG tag–specific antibody (α-FLAG), respectively. The blot is representative of three biological replicates. *B*, FLAG-tagged PKD1 and PKD1^F104E^ were co-expressed with EGFP-tagged PKD1, PKD2, and PKD3 in HEK293T cells. Lysates were probed for expression levels by Western blotting using GFP-specific and FLAG-specific antibodies (α-GFP and α-FLAG, respectively). Co-immunoprecipitate (GFP-trap) was probed with a FLAG-specific antibody. The figure is representative of three independent experiments. *C*, HEK293T cells expressing EGFP-PKD1 constructs were stimulated with 10 μm carbachol for 15 min at 37 °C. Western blotting shows activation loop phosphorylation at Ser^738^/Ser^742^ (α-P-PKD) as well as EGFP-PKD1 expression levels (α-GFP). K612N and D706N are two different kinase-dead versions of PKD. *D*, quantification of *C* by densitometry. Activation loop phosphorylation signal (α-P-PKD) was divided by the total EGFP-PKD1 signal (α-GFP) and normalized to unstimulated WT. Mean and S.D. (*error bars*) are derived from three independent experiments. *E*, NIH3T3 cells expressing the same EGFP-PKD1 constructs as in *C* were stimulated with 1 μm human PDGF-BB (50 ng/ml) for 30 min at 37 °C. PKD activation was assessed by Western blotting using phospho-Ser^738^/Ser^742^–specific antibody (α-P-PKD). Expression levels were probed with GFP-specific antibodies. The blots are representative of three independent experiments.

In the cell, PKD forms homo- and heterodimers, and oligomerization of PKD was previously shown to be dependent on the N terminus of the protein (25, 26). We first tested whether the dimerization interface observed in the crystal structure was required for PKD dimerization in cells. To this end, we introduced the phenylalanine to glutamate point mutation that disrupts the dimer of recombinant *C. elegans* ULD and ULD–C1a proteins in solution (F59E) (Fig. 2, *B* and *C*) into the human PKD1 full-length protein (F104E) and assessed the ability of this protein to form dimers by co-immunoprecipitation. The single point mutation largely abrogated PKD1 dimerization inside cells, whereas deletion of the entire ULD (ΔULD) completely abolished PKD dimerization (Fig. 5*A*). Consistently, heterodimerization between PKD1 and PKD2 or PKD3 was also largely abrogated for the F104E point mutant (Fig. 5*B*), indicating that this interface can mediate PKD homo- and heterodimerization in cells.

PKD is activated by membrane recruitment via the lipid second messenger DAG and subsequent activation loop phosphorylation (20). To test whether ULD dimerization was required for PKD activation, we ectopically expressed different mutants of EGFP-tagged human PKD1 proteins in HEK293T cells and triggered DAG production by adding the muscarinic receptor agonist, and PLC activator, carbachol, which was previously shown to cause PKD activation (4). To assess PKD activation in these cells, we monitored activation loop phosphorylation using an antibody that specifically recognizes phosphorylation of Ser^738^ and Ser^742^ in the activation loop of the kinase domain (Fig. 5, *C* and *D*). WT PKD1 showed basal activation loop phosphorylation that increased about 2.8-fold when carbachol was added. However, PKD1 carrying the monomerizing point mutation in the ULD exhibited lower basal phosphorylation levels that did not increase upon carbachol stimulation, indicating that ULD dimerization is indeed necessary for PKD1 activation. Similarly, PKD1 lacking the entire ULD could also not be as efficiently activated by the addition of carbachol as WT PKD1. This finding suggested a potential role for the ULD in kinase activation by trans-autophosphorylation, a mechanism previously postulated (24), rather than phosphorylation by an upstream kinase. Paradoxically, however, we found that two kinase-dead mutants of PKD1 (K612N and D706N) exhibited similar basal levels of activation loop phosphorylation as compared with WT PKD1 and showed also an increase in activation loop phosphorylation as a consequence of carbachol stimulation. However, this observation can be explained by the heterodimerization of ectopically expressed PKD1 with endogenous (WT) PKD1, PKD2, or PKD3 (25, 26) (Fig. 5*B*), all of which are expressed in HEK293T cells (33). Importantly, when the monomerizing mutation was introduced into the kinase-dead mutants, basal and carbachol-stimulated activation loop phosphorylation were abolished, confirming that dimerization with endogenous PKD most likely gives rise to this effect.

In fibroblasts, PKD can be activated by PDGF stimulation of the PDGF receptor, which leads to DAG production via PLCγ (34). To confirm our findings in a different cellular context, we therefore transfected NIH3T3 cells with EGFP-PKD1 transgenes and stimulated them with PDGF (Fig. 5*E*). Whereas the WT PKD1 protein could be activated by the addition of PDGF, PKD1 carrying the F104E point mutation in the ULD displayed a lower basal activation loop phosphorylation that could not be robustly increased by the addition of PDGF. This observation is very similar to the activation of PKD1 in HEK293T cells stimulated with carbachol (Fig. 5*C*). In summary, these two experiments demonstrate that ULD dimerization is required for PKD activation both in GPCR-mediated and RTK-mediated signaling pathways.

### The ULD stabilizes the autoinhibited conformation of PKDs in cells

**Figure 6.**
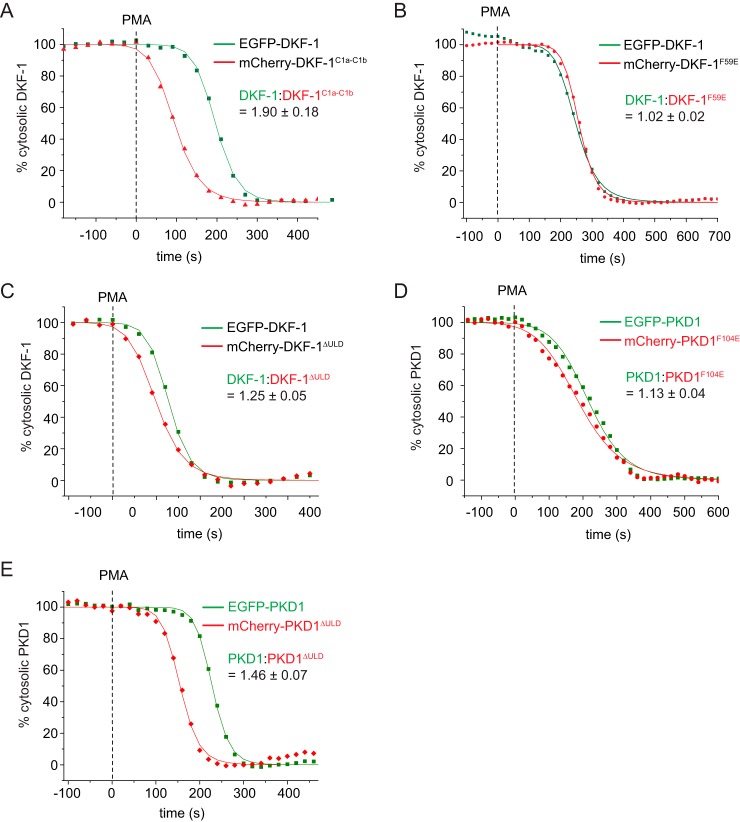
**The ULD stabilizes the autoinhibited conformation of PKDs in cells.** *A–E*, live cell membrane translocation assay. Cytoplasmic fluorescence intensities of COS7 cells co-expressing EGFP- and mCherry-tagged PKD variants measured by confocal microscopy. The fluorescent tags are spectrally separated, and the depletion of cytoplasmic intensity is monitored upon the addition of the plasma membrane ligand PMA at the indicated time points (*squares* or *triangles*). Depletion of cytosolic fluorescence intensity was fitted with a logistic fit function (*line*) to determine half-maximum translocation time. Depicted is one representative translocation curve for WT and mutant PKD proteins from a single cell. The mean ratio of the half-maximum translocation time and the respective S.D. was determined from several individual cells (*n* ≥ 4). *A*, EGFP-tagged full-length DKF-1 (DKF-1^1–722^) was co-expressed with mCherry-tagged C1a-C1b domains (DKF-1^95–239^); *n* = 5. *B*, EGFP-tagged full-length DKF-1 (DKF-1^1–722^) co-expressed with mCherry-tagged DKF-1 carrying the monomerizing point mutation in the ULD (DKF-1^F59E^); *n* = 6. *C*, EGFP-tagged full-length DKF-1 (DKF-1^1–722^) co-expressed with mCherry-tagged DKF-1 lacking the ULD (DKF-1^ΔULD^); *n* = 4. *D*, EGFP-tagged human full-length PKD1 (PKD1^1–912^) co-expressed with mCherry-tagged PKD1 carrying the monomerizing point mutation in the ULD (PKD1^F104E^); *n* = 5. *E*, EGFP-tagged human full-length PKD1 (PKD1^1–912^) co-expressed with mCherry-tagged PKD1 lacking the ULD (PKD1^ΔULD^); *n* = 4.

It has long been thought that, prior to activation, PKD exists in an autoinhibited conformation with low catalytic activity dependent on intramolecular interactions between the membrane-binding C1 and PH domains and the kinase domain (17, 18). We thus investigated whether the ULD might be involved in PKD autoinhibition similar to the C1 and PH domains. To probe the autoinhibited state of PKD in live cells, we employed a membrane translocation assay previously developed for the DAG/phorbol ester–binding PKCs (35). Briefly, this assay measures the relative membrane translocation rates of WT and mutant PKD within a single cell by confocal microscopy. To achieve this, WT and mutant PKD constructs are tagged with spectrally separable fluorescent proteins (EGFP, mCherry), and recruitment to the plasma membrane within a single cell is monitored by cytosolic depletion of PKD upon the addition of an excess of the C1 domain ligand phorbol 12-myristate 13-acetate (PMA). The relative translocation kinetics of a full-length PKD protein and the isolated tandem C1 domains showed that the C1 domains translocate much faster (Fig. 6*A*), consistent with a stable intramolecular assembly in which the DAG-binding sites of the C1 domains are sequestered, as has been observed previously for PKC (35). We then tested whether mutation in the ULD dimerization interface has an effect on the stability of this autoinhibited state, but we observed no determinable difference for DKF-1 (Fig. 6*B*) and only a very subtle difference in membrane translocation kinetics of 1.1-fold for human PKD1 (Fig. 6*D*). In contrast, deletion of the entire ULD led to distinctly accelerated translocation kinetics of DKF-1 (1.3-fold; Fig. 6*C*) and human PKD1 (1.5-fold; Fig. 6*E*). This indicates that the ULD both in DKF-1 and PKD1 serves the function of an autoinhibitory module that stabilizes the closed, cytosolic conformation of the protein. However, because the monomerizing point mutation in the ULD does not accelerate membrane translocation kinetics to the same extent as deletion of the entire ULD, dimerization of the ULD is not required for the stability of this autoinhibitory assembly.

### PKD1 kinase domain can undergo autophosphorylation on Ser^742^ in vitro

**Figure 7.**
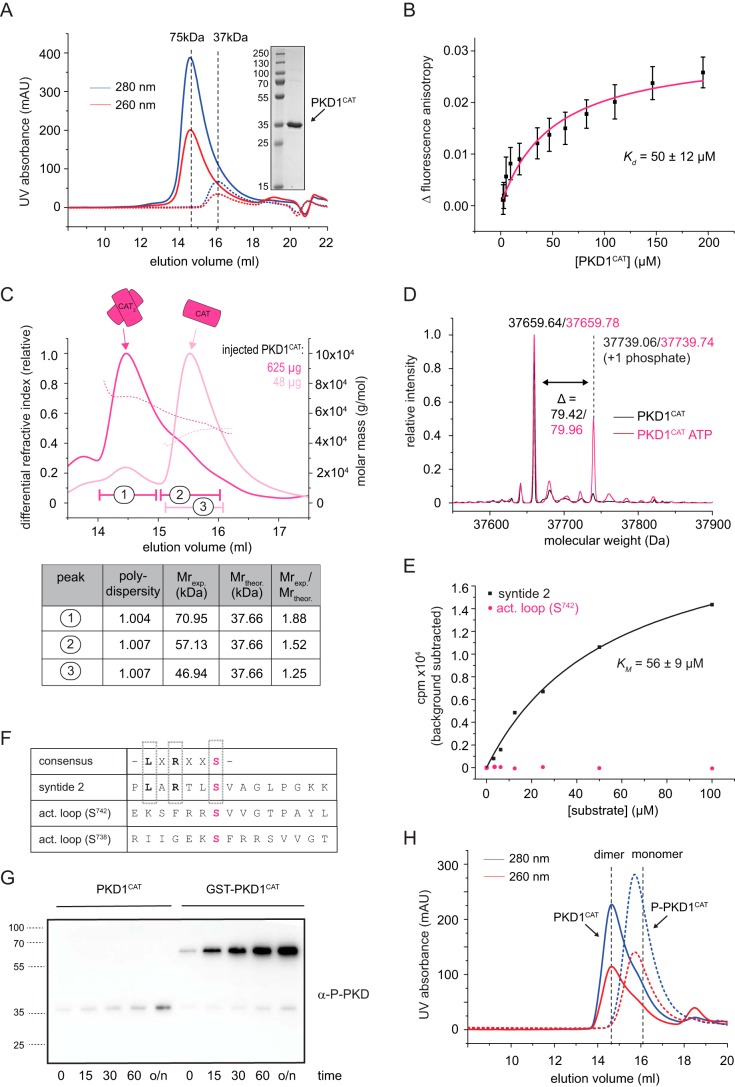
**The PKD1 kinase domain autophosphorylates on its activation loop *in vitro*.** *A*, size-exclusion chromatography (S200 10/300) of the purified kinase domain of PKD1 (PKD1^CAT^). *Inset*, Coomassie-stained SDS-PAGE loaded with a molecular weight marker ranging from 15 to 250 kDa and the pooled and concentrated fractions of the first run (*solid lines*). For this, 500 μl of 3 mg/ml (*solid line*) were injected onto the column. Injecting only 0.3 mg/ml (*dotted line*) shifts the elution volume. *Dashed line*, expected elution volumes for a monomeric (37-kDa) or dimeric (75-kDa) kinase domain derived from calibration of the column with globular molecular weight standards. *B*, fluorescence anisotropy of Atto488-labeled PKD1^CAT^ (80 nm) as a function of increased, unlabeled PKD1^CAT^. The averaged change in fluorescence anisotropy (*black squares*) and the S.D. were derived from 31–60 technical replicates. To derive a *K_d_*, the individual data points were fitted with a single binding mode (*magenta line*). *C*, static light scattering (SEC-MALS) of PKD1^CAT^. The peak fractions of the first run (625 μg, *dark magenta*) were pooled, and the same volume was reinjected onto the system (48 μg, *light magenta*). The *table* summarizes the polydispersity (*Mn*/*Mw*), the experimentally determined molecular weight (*M*_exp._), and the oligomeric state (*M*_exp._/*M*_theor._) for two peaks of the first run (*1* and *2*) and one peak of the second run (*3*). The manually selected peak boundaries are indicated on the chromatogram. *D*, intact MS of purified PKD kinase domain (*black*, PKD1^CAT^) and the same protein incubated at a concentration of 10 μm with 1 mm ATP and 2 mm MgCl_2_ at room temperature overnight (*magenta*, PKD1^CAT^ ATP). *E*, radiometric kinase assay using PKD1^CAT^, [γ-^32^P]ATP, and a conventional CAMK peptide substrate (syntide 2, *black*) or a peptide resembling the activation loop of PKD1 (*act. loop (S^742^)*, *magenta*). The increase in radioactivity was plotted against the substrate concentration. Fitting the data points for syntide 2 to a Michaelis–Menten equation, a *K_m_* of 56 ± 9 μm was derived. The experiment was repeated three times with similar results. *F*, *table* indicates the consensus substrate sequence of PKD, displaying a leucine (*L*) at the −5 position and an arginine (*R*) at the −3 position with respect to the phosphorylatable Ser (or Thr) residue. *X*, any amino acid. The synthetic peptide syntide 2, a CAMK substrate, conforms to the PKD consensus motif. Both phosphorylation sites in the activation loop (Ser^738^ and Ser^742^), however, do not resemble a PKD consensus substrate site. Syntide 2 and the peptide covering amino acids 736–750 of the activation loop of PKD1 (*act. loop (S^742^)*) were used in the radiometric kinase assay. *G*, activation loop phosphorylation investigated by immunoblot using the anti-Ser^738/742^ antibody. 1 μm recombinant PKD1^CAT^ or 1 μm recombinant GST-PKD1^CAT^ was incubated with 1 mm ATP and 2 mm MgCl_2_ at room temperature, and a sample was taken at the indicated time points (0, 15, 30, and 60 min and overnight (*o/n*)), mixed with 2× SDS-loading dye, and immediately boiled at 95 °C. 10 ng of each sample were then subjected to SDS-PAGE and blotted for immunoblot analysis. *H*, size-exclusion profile of PKD1^CAT^ (*solid line*) and stoichiometrically phosphorylated PKD1^CAT^ (P-PKD1^CAT^, *dashed line*). The *black dashed lines* indicate the elution volume expected for a monomeric or dimeric kinase domain and are derived from the calibration of the column with globular protein standards.

To further investigate whether PKD can directly autophosphorylate its activation loop, we purified the recombinant human PKD kinase domain (PKD1^CAT^; Fig. 1*A*) to homogeneity. Size-exclusion chromatography of the recombinant protein suggested a concentration dependent monomer–dimer equilibrium (Fig. 7*A*). Consistently, we observed the transition from the molecular weight of a dimer to that of a monomer by static light scattering (Fig. 7*B*). The *K_d_* of this interaction was determined to be 50 ± 12 μm by fluorescence anisotropy (Fig. 7*C*). We also confirmed the mass and phosphorylation state of this protein by intact MS, which showed the purified kinase domain to be essentially unphosphorylated (Fig. 7*D*). However, when PKD1^CAT^ was incubated with ATP, a second prominent peak, shifted by 80 Da, was visible in the spectrum, indicating the incorporation of one phosphate group. Phosphopeptide mapping of the ATP-incubated protein revealed that the majority of phosphopeptides could be mapped to the Ser^742^ site and a smaller proportion to the Ser^738^ site (Fig. S5*A*), demonstrating that PKD1^CAT^ is able to autophosphorylate on Ser^742^ (and to a lesser extent on Ser^738^) *in vitro*.

Whereas this finding is consistent with the notion that PKD can be activated by trans-autophosphorylation *in vivo* (24) and also with our own activation data (Fig. 5, *C* and *E*), it is notable that the activation loop comprising Ser^742^ (and also Ser^738^) does not conform to the PKD substrate consensus sequence (36). Concerning this matter, we found that the purified PKD1^CAT^ was readily active against syntide 2, a common PKD substrate peptide, but completely inactive against a peptide of the same length corresponding to the activation loop sequence around Ser^742^ (Fig. 7*E*), demonstrating that the activation loop *per se* is a poor substrate for PKD. Furthermore, we noted that PKD autophosphorylation did not appear to be very efficient *in vitro* (Fig. 7*D*). We reasoned that this could be due to the low concentration of 1 μm PKD1^CAT^ used in our experimental setup, which is about 50 times below the dissociation constant of 50 ± 12 μm (Fig. 7*C*). In the context of full-length PKD, ULD-mediated dimerization (Fig. 5*A*) would shift the equilibrium in favor of dimerization of the kinase domain. We therefore hypothesized that additional dimerization increases the efficiency of kinase domain dimerization and subsequently trans-autophosphorylation. We tested this hypothesis by using purified PKD1^CAT^ fused to the dimeric affinity tag GST (GST-PKD1^CAT^). We found that for GST-PKD1^CAT^, the extent of PKD autophosphorylation was substantially higher than for PKD1^CAT^ (Fig. 7*F*), showing that additional dimerization increases the autophosphorylation rate of PKD1^CAT^.

We then took advantage of this observation to generate stoichiometrically phosphorylated PKD1^CAT^ (P-PKD1^CAT^) (see “Experimental procedures”). Intact MS showed that P-PKD1^CAT^ was mostly monophosphorylated, and phosphopeptide mapping revealed that Ser^742^ was the predominant phosphorylation site of this protein (Fig. S5*B*), confirming that autophosphorylation is primarily carried out on Ser^742^. Finally, we observed that P-PKD1^CAT^ is monomeric at the same concentration as dimeric PKD1^CAT^ as judged by its elution behavior in SEC (Fig. 7*G*), suggesting that autophosphorylation prohibits dimerization.

In summary, we have observed that the PKD1 kinase domain undergoes activation loop autophosphorylation *in vitro* on Ser^742^, which is substantially enhanced by additional dimerization of the kinase domain. Phosphorylation on Ser^742^ renders the kinase domain monomeric.

## Discussion

We have determined the crystal structure of the ULD–C1a of the *C. elegans* PKD homolog DKF-1. The structure shows that the ULD forms a symmetric dimeric assembly, which is mediated by a hydrophobic patch. We confirmed that this conserved dimerization interface on the ULD is required for dimerization of the recombinant ULD in solution and for dimerization of full-length human PKD1 in cells. Furthermore, we found that the ULD regulates PKD activation on two levels *in vivo*. On the one hand, the ULD stabilizes the autoinhibited conformation of PKD in the cytosol, and on the other hand, dimerization-deficient PKD1 cannot be phosphorylated on its activation loop, indicating that PKD activation requires dimerization and that this is driven, primarily, by the ULD. Finally, we provide evidence that the recombinant PKD1 kinase domain trans-autophosphorylates on Ser^742^ in the activation loop *in vitro*.

**Figure 8.**
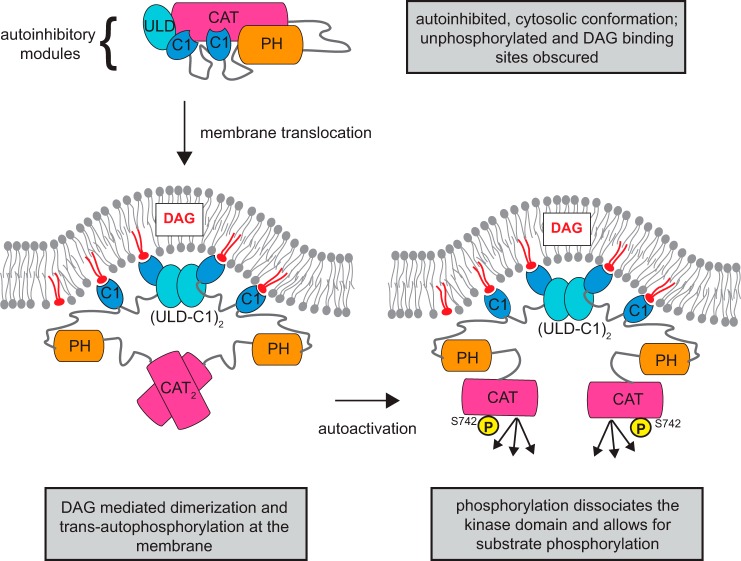
**Model of PKD autoactivation by ULD dimerization.** PKD transitions from an autoinhibitory cytosolic conformation to an active DAG-bound conformation. *Arrows* indicate the sequence of events in this proposed activation mechanism. In the cytosol, the autoinhibitory conformation is maintained by the regulatory ULD, C1, and PH domains. This conformation is presumably unphosphorylated and monomeric, and the DAG-binding sites of the C1 domains are sequestered in this autoinhibitory conformation. When DAG is generated, PKD translocates to the membrane. The engagement of the C1a and C1b to DAG releases the autoinhibition of the kinase domain. Also, the increase in local concentration leads to dimerization of the ULD, which facilitates kinase domain dimerization and trans-autophosphorylation in the activation loop. Ultimately, phosphorylation of Ser^742^ breaks the dimeric arrangement of the kinase domains, which allows the kinase domains to engage and phosphorylate the substrates.

Based on these findings, we propose a new model for PKD activation in which PKD activation loop phosphorylation is not governed by upstream kinases such as nPKC but relies on dimerization mediated by its ULD and subsequent autophosphorylation (Fig. 8). We suggest that, prior to activation, PKD exists in a cytosolic, autoinhibited, and presumably monomeric conformation, in which the activation loop is unphosphorylated and the DAG-binding sites of the C1 domains are sequestered in the intramolecular assembly. Upon the production of DAG, PKD is recruited to DAG-containing membranes, thereby relieving autoinhibition. In addition, membrane binding serves to locally concentrate PKD on the membrane, thereby promoting ULD-mediated dimerization. The physical association of two PKD molecules via their ULDs also increases the propensity of their related kinase domains to dimerize, which ultimately leads to trans-autophosphorylation on Ser^742^, whereby one kinase domain catalyzes the incorporation of one phosphate group into the other kinase domain. Finally, phosphorylated Ser^742^ causes the dissociation of the kinase domains, which thereafter are competent to engage and phosphorylate their substrates.

This model of concentration-dependent dimerization and autophosphorylation of PKD is in contrast to the view that PKD is activated by upstream kinases (22), but it also contradicts the perception that PKD is predominantly dimeric in cells (26). Concerning the latter, we found that the ULD and kinase domain of human PKD1 homodimerize with dissociation constants of 1.7 ± 0.2 and 50 ± 12 μm, respectively, and that the cellular concentration of human PKD1 in HEK293T cells and rat PKD1 in INS-1 cells is in the mid-nanomolar range. It therefore seems unlikely that PKD is constitutively dimeric in cells. The structure of the DKF-1 ULD–C1a shows that dimerization is mediated by a relatively small patch of mostly hydrophobic amino acids on the surface and that this undergoes a transition to a monomeric species at concentrations approaching those determined *in vivo*. The conclusion that inactive PKD is predominantly monomeric in cells is supported by the predominantly cytoplasmic distribution of PKD (37). However, upon recruitment of PKD to a specific subcellular compartment, its concentration will be substantially higher, and this presumably allows for efficient dimerization and autophosphorylation. In this respect, PKD localizes and is reportedly active at the outer membrane of the TGN (2, 15), which is a small two-dimensional compartment compared with the large volume of the cytoplasm. In general, the engagement with DAG restricts the diffusion of PKD to the plane of the membrane, which will additionally increase its local concentration and would be expected to trigger dimerization, trans-autophosphorylation, and activation.

At this point, we cannot exclude the possibility that other regulatory domains within PKD, such as the C1a, C1b, or PH domain, or other cellular factors contribute to dimerization of PKD. However, it is clear that introducing a single point mutation in the dimerization interface of the ULD is sufficient to abolish co-immunoprecipitation of full-length PKDs. Because we also found that the kinase domain of PKD dimerizes *in vitro*, one could speculate that the ULD and kinase domain work synergistically to constitutively dimerize PKD *in vivo*. However, we do not think that this is a very likely scenario. Given our observations that a constitutively dimeric GST kinase domain fusion shows rapid activation loop autophosphorylation and that this autophosphorylated kinase domain can no longer dimerize, it seems most likely that dimerization of the PKD kinase domain only occurs transiently and serves the purpose of mediating trans-autophosphorylation rather than mediating a stable dimeric assembly of PKD. Importantly, ULD dimerization–deficient PKD1 does not get phosphorylated in cells in response to DAG production, suggesting that PKD trans-autophosphorylation requires the ULD for efficient dimerization.

Dimerization and activation by autophosphorylation are key features of many kinases and occur via distinct mechanisms. The most closely related kinase to PKD within the family of CAMK kinases is checkpoint kinase 2 (Chk2). Intriguingly, Chk2 is also activated by dimerization and trans-autophosphorylation, which was corroborated by crystal structures that trapped Chk2 in dimeric conformations. In these structures, the activation loop of one kinase domain is in close proximity to the catalytic cleft of the other kinase domain, thereby permitting the trans-phosphorylation of one activation loop by the neighboring kinase domain (38, 39). An interesting parallel between Chk2 and PKD is that the autophosphorylation site(s) in their activation loops does not conform to the substrate consensus site of these kinases. It has therefore been proposed that Chk2 autophosphorylation is mechanistically different from Chk2 substrate phosphorylation and that autophosphorylation requires an intimate dimeric arrangement of the kinase domains and regulatory domains (40). Our data suggest that this may also be the case for PKD. We show that PKD1^CAT^ can readily phosphorylate syntide 2, a consensus substrate peptide, but is inactive against a peptide comprising the activation loop sequence. However, we observed that the same activation loop motif is phosphorylated when the whole kinase domain is presented as a substrate and that the catalytic efficiency of this trans-autophosphorylation reaction can be increased when the kinase domains are additionally dimerized by a GST tag. This indicates that, as in Chk2, the PKD kinase domain requires additional dimerization for efficient activation loop autophosphorylation. However, PKD and Chk2 are only related within their kinase domains, and the regulatory domains of PKD, including the ULD, are not shared with Chk2, which only contains an N-terminal SQ/TQ-cluster region followed by an FHA domain. It has been reported that phosphorylation of Thr^68^ in the SQ/TQ-cluster by the upstream kinase ATM dimerizes Chk2 via phospho-Thr^68^–FHA domain interactions, which is required for efficient trans-autophosphorylation (41) of the FHA–kinase domain dimer (39), although autophosphorylation was also detected for the isolated kinase domain, albeit less efficient (38). We therefore hypothesize that PKD and Chk2, despite their differences in regulatory domains and activating stimuli, share an overall similar mechanism of activation in which the phospho-Thr^68^–FHA–mediated dimerization in Chk2 is functionally equivalent to the DAG-induced ULD dimerization in PKD. In both cases, dimerization via the regulatory domains serves the function of mediating a dimeric assembly of the kinase domains, which allows for efficient trans-autophosphorylation at a nonconsensus site in the activation loop.

Such a mechanism calls into question the necessity of upstream kinases for PKD activation. Whereas early reports proposed that activation loop phosphorylation of PKD was carried out by novel PKCs (22), more recent studies have demonstrated PKC-independent activation loop phosphorylation of PKD *in vivo* upon Gα_q_-PLCβ DAG production (24) and demonstrated its requirement for mitogenesis (3). These reports found that *in vivo* autophosphorylation in the PKD activation loop is primarily on Ser^742^ and to a minor extent on Ser^738^, which is consistent with our finding that the isolated PKD1 kinase domain undergoes autophosphorylation primarily at Ser^742^ and much less efficiently at Ser^738^ *in vitro*. Of particular interest is the fact that we did not observe detectable amounts of doubly phosphorylated kinase domain in this experiment, which indicates that phosphorylation of one site (predominantly Ser^742^) may preclude autophosphorylation of the other site. Furthermore, the position of the predominant autophosphorylation site Ser^742^ corresponds to the well-established canonical phosphorylation site observed in many other kinases (42). Given our observations and the fact that Ser^742^ does not conform to a PKC consensus motif, we think that it is unlikely that novel PKCs directly trans-phosphorylate PKD on Ser^742^. However, we cannot rule out the possibility that PKCs are involved in the regulation of PKD activity either indirectly or via phosphorylation of alternative sites, including Ser^738^, although it should be noted that this does not conform to a PKC consensus sequence either (43). A recent study that investigated the PKD-dependent activation of the NLRP3 inflammasome showed that whereas three PKD-specific inhibitors were able to block the activation of the NLRP3 inflammasome, a PKC-specific inhibitor (Gö6983) did not have any effect on NLRP3 activation, suggesting that PKC activity is dispensable for this PKD-dependent process (5).

Whereas trans-phosphorylation by novel PKCs seems questionable, PKD may be trans-phosphorylated by other PKD isoforms. Previous studies have reported that PKD isoforms form heterodimers and that the ability to heterodimerize depends on the N terminus of the protein (25, 26). This is consistent with our own findings and explained by the fact that the ULD dimerization interface is identical in the three human PKD isoforms. Together, these data suggest that ULD-mediated dimerization may permit heterodimerization of PKD isoforms in the cell, although the identification of heterodimers from ectopic overexpression of one or more isoforms should be treated with caution. What is not known is whether PKD heterodimers are functional; trans-isoform phosphorylation would also be dependent on kinase domain heterodimerization, which will require further investigation. Nevertheless, the dominant-negative phenotypes previously reported (2, 26) may be attributable to ULD-mediated heterodimerization of PKD that occurs during ectopic overexpression of a single isoform.

Beyond its role in PKD activation, we provide evidence that the ULD stabilizes the inactive, cytosolic conformation of PKD. This conclusion can be drawn from the fact that deletion of the ULD results in enhanced accessibility of the membrane binding C1 domains in cells. The finding that the F59E mutation in DKF-1 does not accelerate membrane translocation and that the equivalent F104E mutation in PKD1 only slightly accelerates membrane translocation, whereas deletion of the entire domain significantly accelerates membrane translocation, suggests that dimerization of the ULD does not account for this stabilizing effect. This assay further confirms that the DAG-binding sites of the C1 domains are sequestered in the cytosolic inactive conformation of PKD. Whereas the molecular details of this autoinhibited conformation of PKD remain to be determined, it seems likely that each regulatory domain (PH, C1b, C1a, and ULD) contributes to the stability of this assembly because deletion of any of the domains either partially activates PKD (17, 18) or accelerates the membrane association of PKD with phorbol esters. In this context, the close apposition of the ULD with the C1a domain and the dynamic interaction between these domains could be of great importance; one possible function of the ULD–C1a module could be to couple membrane engagement of the C1 domain to the displacement of the ULD from the kinase domain analogous to the pseudosubstrate-C1a module in PKC (35).

An alternative, and not mutually exclusive, explanation for the evolution of the ULD–C1a as a functional unit could be that it enables recognition of membranes of particular architecture. The arrangement of the C1a domains observed in the crystal structure would be incompatible with PKD binding to a flat membrane, because the DAG-binding clefts point almost diametrically opposite to each other. Our biophysical characterization showed that in solution, the orientation of the membrane binding sites of the C1a domains is not that rigid, but also not completely random, but rather samples a limited conformational space. This might allow PKD to detect DAG in specific membranes, namely those with a high degree of negative curvature. Sensing DAG in the right membrane context might be particularly important for the role of PKD in the fission of nascent TGN-derived vesicles, which happens in a dynamic membrane environment that is characterized by high DAG levels and negative membrane curvature (2, 44) and that is reportedly sensed by the C1a domain of PKD (15). The orientation of the C1a domains is restricted not only by the dynamic interaction between the C1a domain and the ULD, but also by the interaction between the two ULD protomers. Therefore, it will be important to determine whether dimerization of the ULD is required for the correct subcellular localization of the C1a domains or for the entire PKD protein.

In summary, we provide the first structural insight into how PKD dimerizes using a novel and previously unannotated ULD. Our results show that this mode of dimerization is required for PKD activation loop phosphorylation, a critical step in PKD activation, and we therefore propose a new mechanism for PKD activation. In this model, PKD activation is achieved via DAG-driven ULD-mediated dimerization and kinase domain trans-autophosphorylation.

## Experimental procedures

### Protein expression and purification

The ULD of *C. elegans* DKF-1 (DKF-1^1–94^), human PKD1 (PKD1^48–143^), and human PKD3 (PKD3^55–149^) as well as the ULD–C1a of *C. elegans* DKF-1 (DKF-1^1–151^) and PKD3 (PKD3^55–206^) were cloned into the pGST parallel vector, resulting in an N-terminally tagged GST fusion protein. Human PKD1 ULD–C1a (PKD1^48–198^) and human PKD2 (PKD2^42–190^) were cloned into pET-28a vector, resulting in a C-terminal His_6_ tag. Plasmids were transformed into BL21* *Escherichia coli*, expression was induced by the addition of 200 μm isopropyl 1-thio-β-d-galactopyranoside, and proteins were expressed for 20 h at 20 °C. For ^15^N isotope labeling, DKF-1^1–94^ and DKF-1^1–151^ were expressed in M9 minimal medium containing 1 g/liter ^15^NH_4_Cl. For ^15^N and ^13^C labeling, DKF-1^1–94^ was expressed in M9 medium containing 1 g/liter ^15^NH_4_Cl and 5 g/liter ^13^C_6_ d-glucose (both from Eurisotopes). Cell pellets from *E. coli* were dissolved in 50 ml of lysis buffer (50 mm Tris, pH 7.5, 150 mm KCl, 2 mm MgCl_2_, 2 mm benzamidine, 1 mm TCEP), and lysates were incubated with 10 mg of lysozyme, 1 μl of benzonase for 30 min on ice and subsequently sonicated, and cell debris was removed by centrifugation. Proteins were then purified from cleared lysates by affinity chromatography using GSH beads (GE Healthcare). The GST tag was cleaved off on the beads using 0.02 mg/ml TEV protease (generated in-house), and proteins were finally buffer-exchanged by size-exclusion chromatography (S75 16 60, GE Healthcare) into SEC buffer (20 mm Tris, pH 7.5, 150 mm KCl, 1 mm TCEP), frozen in liquid nitrogen, and stored at −80°. Mutants of the DKF-1 ULD (ULDF^F59E^, ULD–C1a^F59E^, ULD^R42C^) were generated by site-directed mutagenesis, and proteins were expressed and purified in the same way as their WT counterparts.

GST-tagged PKD1^CAT^ (residues 569–892) was expressed using baculovirus-infected Sf9 insect cells. Cell pellets were lysed in lysis buffer (50 mm Tris, pH 7.5, 150 mm KCl, 0.5% CHAPS, 1 mm TCEP, 2 mm MgCl_2_, 1 mm phenylmethylsulfonyl fluoride, and protease inhibitors (Sigma)) for 1 h, and the lysate was cleared by centrifugation at 38,400 × *g*. The clarified lysate was incubated with GSH beads equilibrated in washing buffer (50 mm Tris, pH 7.5, 150 mm KCl, 0.25% CHAPS, 1 mm TCEP) for 2 h at 4 °C under constant rotation. GST-bound protein was washed with high-salt buffer (300 mm KCl) and low-salt buffer (60 mm KCl). 0.02 μg/ml TEV protease was added for overnight cleavage to remove the tag. To obtain GST-fused PKD1^CAT^, the protein was eluted from the beads with GSH (50 mm Tris, pH 7.5, 60 mm KCl, 0.25% CHAPS, 1 mm TCEP, 10 mm GSH). PKD1^CAT^ or GST-PKD1^CAT^ was then loaded onto a HiTrap Q FF column (GE Healthcare) equilibrated in Q_A_ buffer (50 mm Tris, pH 7.5, 1% glycerol, 1 mm TCEP) and eluted with a gradient of 0–1 m KCl. Peak fractions were collected, concentrated, and further purified by size-exclusion chromatography, using a S200 10/300 column (GE Healthcare) equilibrated in 20 mm Tris, pH 7.5, 150 mm KCl, and 1 mm TCEP. Stoichiometrically phosphorylated PKD1^CAT^ (P-PKD1^CAT^) was generated by resuspending GST-PKD1^CAT^–bound GSH beads in autophosphorylation buffer (20 mm Tris, pH 7.5, 150 mm KCl, 1 mm TCEP, 0.25% CHAPS, 10 mm ATP, 20 mm MgCl_2_). Beads were incubated for 100 min at room temperature followed by 14 h at 4 °C. The GST tag was subsequently removed by TEV cleavage, and the hyperphosphorylated PKD1^CAT^ was loaded onto a high-resolution MonoQ column (GE Healthcare), equilibrated in MonoQ_A_ buffer (20 mm Tris, pH 8.5, 1% glycerol, 1 mm TCEP). The different PKD1^CAT^ phosphospecies were separated by a linear gradient ranging from 0 to 50% MonoQ_B_ buffer (20 mm Tris, pH 8.5, 1 m KCl, 1% glycerol, and 1 mm TCEP) over 50 column volumes. The peak fractions corresponding to the monophosphorylated protein were concentrated and further purified by S200 10/300 as for PKD1^CAT^. P-PKD1^CAT^ was validated by intact MS (Fig. S3) and shown to be primarily monophosphorylated. PKD1^PH-CAT^ (comprising residues 395–892), was expressed as a GST fusion in Sf9 cells and purified by GSH beads like PKD1^CAT^. TEV cleavage of PKD1^PH-CAT^ was carried out in 50 mm HEPES, pH 7.0, 300 mm KCl, and 1 mm TCEP. After TEV cleavage, (NH_4_)_2_SO_4_ was added to a final concentration of 1.3 m. Precipitate was pelleted by centrifugation. The supernatant was filtered and loaded onto an HIC column (HiTrap Phenyl FF, GE Healthcare) washed with HIC_A_ buffer (50 mm HEPES, pH 7.0, 1.3 m (NH_4_)_2_SO_4_, 1 mm TCEP) and eluted with a linear gradient from 0 to 100% HIC_B_ buffer (50 mm HEPES, pH 7.0, 1 mm TCEP). Finally, PKD1^PH-CAT^ was gel-filtered by S200 10/300 into 20 mm HEPES, pH 7.0, 300 mm KCl, 1 mm TCEP.

The S200 10/300 column was calibrated using the HMW gel filtration calibration kit (GE Healthcare) according to the manufacturer's instructions. In addition to the proteins from this kit, chymotrypsin (25 kDa) was included into the calibration.

### Crystallization

Crystals were set up as sitting drops in a 48-well plate (MRC Maxi 48-Well, Hampton Research) by mixing 1 μl of purified DKF-1^ULD–C1a^ (5.6 mg/ml) and 1 μl of reservoir solution (6.8% (w/v) PEG8K, 18% (v/v) ethylene glycol, 0.1 m MES, pH 6.5, 0.03 m NaF, 0.03 m NaB, 0.03 m NaI) and grew within 3 days at room temperature. Crystals were cryoprotected by soaking them for 60 s in 15% (w/v) PEG8K, 30% ethylene glycol, 0.03 m NaF, 0.03 m NaB, 0.03 m NaI, 0.1 m MES, pH 6.5, and frozen in liquid nitrogen.

### Data collection and structure determination

Diffraction data were collected at the European Synchrotron Radiation Facility (ESRF), Grenoble, France. A native data set was collected to 2.3 Å resolution on ID29. The structure was phased by single anomalous dispersion in ShelXD using data collected at the zinc edge (λ = 1.28 Å). A model for residues 12–95 was built into the electron density visible in the initial maps. The C1 domain of PKCδ (PDB entry 1PTQ) was positioned in the remaining electron density using the positions of the zinc atoms obtained from the anomalous difference map, followed by manual rotation, morph-fit, and real space refinement in Coot. Restrained refinement of the resulting model in Refmac was sufficient to reduce the *R*-factors to *R* = 33%/*R*_free_ = 36%, which was good enough for automated model building in Buccaneer. The final model comprises residues 12–151 with *R* = 20.1 and *R*_free_ = 26.8.

### Static light scattering

Monodispersity and oligomeric state were assessed by size-exclusion chromatography on a Superdex 200 10/300 (GE Healthcare) operated by a 1260 Infinity HPLC (Agilent Technologies) system coupled to MALS. Light scattering was detected by a MiniDawn Treos (Wyatt) detector equipped with a 690-nm laser, and the refractive index was measured by a Shodex RI-101 (Shodex) detector. The column was extensively equilibrated in gel filtration buffer (20 mm Tris, pH 7.5, 150 mm KCl, 1 mm TCEP), and 50 μl of 2.5 mg/ml of protein were injected for each analysis.

### Protein co-expression and pulldown

PKD3^55–206^ with an N-terminal GST tag and PKD1^48–198^ and PKD2 (PKD2^42–190^) with a C-terminal His_6_ tag were (co-)transformed into BL21*. Proteins were (co-)expressed and lysed, and GSH affinity purification was carried out as described above. The cleared lysate and GSH beads were probed for containing proteins by Western blotting using anti-GST antibody (anti-GST (B-14), Santa Cruz Biotechnology, Inc.) and anti-His antibody (THE^TM^ anti-His, GenScript) as recommended by the supplier.

### Mass spectrometry

For intact protein MS, proteins were diluted in 0.1% formic acid (FA) to a concentration of 20 ng/μl, and 100 ng were loaded on an Aeris Widepore C4 column, 3.6-μm particle size, dimensions 2.1 × 150 mm (Phenomenex), using a Dionex Ultimate 3000 HPLC system (Thermo Scientific) with a working temperature of 55 °C: 0.1% FA as solvent A; 90% acetonitrile, 0.08% FA as solvent B. The proteins were separated on a 6-min gradient from 10 to 70% solvent B at a flow rate of 300 μl/min. Mass spectra were recorded on a Waters Synapt G2-Si equipped with a ZSpray ESI source. Glu[1]-Fibrinopeptide B (Glu-Fib) was used as a lock mass, and spectra were corrected on the fly. Data were analyzed in MassLynx version 4.1 using the MaxEnt 1 process to reconstruct the uncharged average protein mass.

The experimental details of peptide MS and phosphopeptide mapping of PKD1 and PKD1 quantification by parallel reaction monitoring are described in the supporting Experimental procedures.

### PKD1^CAT^ autophosphorylation in vitro

10 μm PKD1^CAT^ in 50 mm Tris, pH 7.5, 150 mm KCl, 1 mm TCEP was incubated with 1 mm ATP and 2 mm MgCl_2_ at room temperature overnight.

### Small-angle X-ray scattering

SAXS data were collected for the ULD–C1a construct of DKF-1 on BioSAXS beamline BM29, ESRF using an online size-exclusion chromatography setup. DKF-1^1–151^ was applied to a Superdex 200 column equilibrated in 20 mm Tris, pH 8.0, 100 mm NaCl, and 1 mm TCEP. Images were acquired every second for the duration of the size-exclusion run. Buffer subtraction was performed by averaging 50 frames either side of the peak. Data reduction and analysis was performed using the BsxCuBE data collection software and the ATSAS package (32). The program AutoGNOM (45) was used to generate the pair distribution function (*P*(*r*)) for each isoform and to determine *D*_max_ and *R_g_* from the scattering curves (*I*(*q*) *versus q*) in an automatic, unbiased manner. The crystal structure of dimeric DKF-1^1–151^ was compared with the solution scattering using CRYSOL (46). To model the scattering data with two flexibly linked domains rather than the intramolecular assembly observed in the crystal lattice, we employed rigid-body modeling in CORAL (32). The ULD was defined as amino acids 12–95 and the C1a domain as amino acids 96–149. Missing residues at the N terminus (amino acids 1–11) were implemented as dummy residues. Iterative runs of CORAL were performed in which the position of the ULD domain was fixed, whereas the C1a domain was allowed to move.

### NMR

Standard NMR spectra were recorded at 298 K on a Bruker Avance HD3 + 600- and 800-MHz spectrometer, equipped with room-temperature triple resonance probes with *z*-gradients. To assign the HSQC ^15^N-^1^H cross-peaks, a combination of HNCAB, HNcoCACB, HNCA, and HNcoCA experiments was carried out.

### N-terminal FITC labeling

DKF-1^1–151^ was N-terminally labeled using Sortase A and an FITC-conjugated labeling peptide (LPETGG, GenScript) as described previously (47). Briefly, the reaction was carried out with 50 μm DKF-1^1–151^, 75 μm SrtA (generated in-house with an N-terminal His tag fusion), and an excess of 500 μm FITC-conjugated labeling peptide in labeling buffer (50 mm Tris, pH 7.5, 150 mm NaCl, 10 mm CaCl_2_, 1 mm TCEP) and incubated for 3 h at room temperature. Unbound dye and SrtA were removed by applying the reaction to a nickel-nitrilotriacetic acid affinity column (HisTrap, GE Healthcare) and subsequent size-exclusion chromatography (Superdex 75, 10/300, GE Healthcare).

### Atto488 labeling and fluorescence anisotropy

Human PKD1^ULD^ and PKD1^CAT^ were covalently labeled on their surface-exposed cysteines with a maleimide-conjugated Atto488 dye (ATTO-TEC) according to the manufacturer's instructions. For the *C. elegans* ULD, the surface-exposed arginine 42 was mutated to cysteine. Recombinant DKF-1 ULD^R42C^ was then site-specifically labeled with Atto488, like human PKD1^ULD^, and PKD1^CAT^. In all cases, the unbound dye was separated from the protein by applying the reaction onto a S75 10/300 gel filtration column (GE Healthcare). Labeling efficiency was estimated by extinction coefficient to be ∼80% for ULD^R42C^, 50% for PKD1^ULD^, and 11% for PKD1^CAT^. Fluorescence anisotropy was measured in an PerkinElmer Life Sciences LS50B fluorimeter (λ_ex_ = 505 nm, λ_em_ = 520 nm) at 20 °C. Atto488-ULD^R42C^ was diluted to 35–70 nm in 20 mm Tris, pH 7.5, 150 mm KCl, 1% glycerol, 1 mm TCEP, and ULD or ULD^F59E^ was titrated into it from a 222 or 150 μm stock, respectively. For PKD1^ULD^, Atto488-labeled ULD was diluted to 40 nm in 20 mm Tris, pH 7.5, 150 mm KCl, 1 mm TCEP, 1% glycerol, and unlabeled ULD was titrated to this solution from an 800 μm stock. For PKD1^CAT^, a 195 μm PKD1^CAT^ solution (buffer: 50 mm Tris, pH 7.5, 220 mm KCl, 1% glycerol, 1 mm TCEP) containing 80 nm Atto488-labeled PKD1^CAT^ was measured and successively diluted with buffer containing 80 nm Atto488-labeled PKD1^CAT^. 26–63 measurements were made for each data point with an integration time of 2 s. The change in anisotropy was plotted against the concentration of ULD, ULD^F59E^, PKD1^ULD^, or PKD1^CAT^, and the data were fitted to a single binding model with *y* = *y*_max_ × [ULD]/(*K_d_* + [ULD]) and *y* = *y*_max_ × [CAT]/(*K_d_* + [CAT]), respectively, where y_max_ is the maximum change in anisotropy; [ULD] is the concentration of titrated, unlabeled ULD, ULD^F59E^, or PKD1^ULD^; [CAT] is the concentration of titrated, unlabeled PKD1^CAT^; and *K_d_* is the dissociation constant of the homodimer.

### Cell culture and transfection vectors

COS7, HEK293T, and NIH3T3 cells were cultured in Dulbecco's modified Eagle's medium high glucose (PAA Laboratories), supplemented with 2 mm l-glutamine, streptomycin-penicillin (PAA Laboratories), and 10% FBS (PAA Laboratories), and split every 2–3 days. For transfection of DKF-1 or human PKD1 into mammalian cells, WT and mutant isoforms were cloned into the EGFP-C1 and mCherry-C1 vector (Clontech), resulting in N-terminal protein fusions.

### Co-immunoprecipitation

HEK cells were seeded to 6-well plates and transfected after 24 h with 1 μg of DNA and 4 μl of TurboFect (Thermo Fisher Scientific) per well. After 24 h, cells were detached by trypsinization (200 μl of trypsin) and resuspended with 800 μl of Dulbecco's modified Eagle's medium. Cells were spun down at 4 °C, 1000 × *g* for 5 min and resuspended in 150 μl of lysis buffer (50 mm Tris, pH 7.5, 150 mm KCl, 1 mm MgCl_2_, 0.5% CHAPS, 1 mm TCEP, 2 mm benzamidine, 1× protease inhibitor mixture (Sigma), 1 mm phenylmethylsulfonyl fluoride). Cells were frozen in liquid nitrogen and thawed on ice. After thawing, 1 μl of diluted benzonase (EMD Millipore) (diluted 1:20 in lysis buffer) was added to each lysate and incubated for 40 min on ice. Debris was removed by centrifugation for 20 min, 16,000 × g at 4 °C. 10 μl of supernatant was taken off (input) and probed for PKD transgene expression by Western blotting using the anti-PKD antibody (catalog no. 2052, Cell Signaling). 130 μl of the remainder were applied to either one well of a GFP-trap 96-well plate or mixed with 2 μl of GFP-trap magnetic bead slurry (ChromoTek), both equilibrated with lysis buffer. Beads/wells were washed at least three times with wash buffer (50 mm Tris, pH 7.5, 150 mm KCl, 1 mm TCEP, 2.5% glycerol) using 100 μl/96-well plate and 300 μl for magnetic beads, respectively. Finally, protein was eluted with 100 μl of 2× SDS-loading dye according to the manufacturer's instructions and subjected to immunoblot analysis (GFP-trap elution). Co-immunoprecipitated proteins were probed by using an anti-FLAG antibody (M2, Sigma).

### Western blotting

After electrophoresis, SDS-polyacrylamide gels were blotted onto nitrocellulose membranes (Amersham Biosciences Protran, 0.2 μm) by wet transfer (90 min at a constant voltage of 60 V; buffer: 25 mm Tris, 192 mm glycine, 20% methanol). Membrane was consequently blocked by incubation with TBS-T containing 5% BSA at room temperature for 1 h. If not explicitly stated otherwise by the manufacturer, primary antibodies were diluted 1:1000 in TBS-T containing 5% BSA and incubated for 1 h at room temperature (anti-GST, anti-His) or overnight at 4 °C (anti-FLAG, anti-PKD, anti-phospho-Ser^738/742^). Blot was washed three times for a minimum of 5 min with TBS-T. The blot was then incubated with secondary antibody (anti-rabbit or anti-mouse horseradish peroxidase–conjugated antibody, New England Biolabs), diluted 1:5000 in TSB-T for 1 h at room temperature, and finally washed three times with TBS-T. Chemiluminescence was readout on a Fusion FX Vilber Lourmat (PeqLab) using ECL Select Western Blotting Detection Reagent (Amersham Biosciences, GE Healthcare). Exposure was manually adjusted to avoid saturation of pixels.

### Activation loop phosphorylation in HEK293T and NIH3T3 cells

HEK293T cells were seeded to a 24-well dish and transfected the next day using 500 ng of DNA and 1 μl of TurboFect (Thermo Fisher Scientific) per well. NIH3T3 cells were seeded into a 24-well dish and transfected the next day using 500 ng of DNA and 1 μl of Lipofectamine 3000 (Invitrogen) according to the manufacturer's instructions. Cells were grown for 20 h and showed similar fluorescence prior to stimulation. Medium was taken off, and cells were washed once with HBSS. HEK293T cells were then stimulated with 10 μm carbachol (Sigma) in HBSS at 37 °C. After 15 min, HBSS was completely removed, and the plate was transferred to ice and immediately lysed by adding 100 μl of ice-cold lysis buffer (10 mm Tris, pH 7.5, 225 mm NaCl, 0.1% SDS, 1% Triton X-100, 0.5% sodium deoxycholate, 0.5 mm EDTA, 50 mm NaF, 0.2 mm sodium orthovanadate, 10 mm β-glycerophosphate, 2 mm sodium pyrophosphate, 2 mm benzamidine, 1× protease inhibitor mixture (Sigma), 1 mm TCEP) to each well. Cell lysate was transferred to Eppendorf tubes and vortexed and then frozen in liquid nitrogen. After thawing lysates on ice, 1 μl of a diluted benzonase (EMD Millipore) solution (1:20 diluted in 20 mm Tris, pH 7.5, 150 mm KCl, 100 mm MgCl_2_, 1 mm TCEP) was added to degrade DNA for 30 min. Lysates were spun down at 4 °C on a table top centrifuge at 16,000 × *g* for 20 min, and supernatant was mixed with SDS-loading dye and subjected to SDS-PAGE and Western blotting. Phospho-PKD levels were probed using a phospho-Ser^738^/Ser^742^–specific antibody (catalog no. 2054, Cell Signaling) and EGFP transgene levels using a GFP-specific antibody on two individual blots, and the signals were quantified by densitometry with ImageJ. Transfected NIH3T3 cells were stimulated with 1 μm (50 ng/ml) recombinant human PDGF-BB (BioLegend) diluted in HBSS for 30 min at 37 °C. Lysis and Western blotting was carried out as for HEK293T cells.

### Translocation assay

COS7 cells were seeded into a 4-well chamber microscopy dish (InVitro Scientific) and co-transfected about 20 h after seeding with 0.25 μg of DNA per well and construct using Lipofectamine 2000 (Invitrogen) or TurboFect (Thermo Scientific) according to the manufacturer's instructions. After 24 h, medium was exchanged to HBSS (Life Technologies), and cells were imaged in HBSS on a Zeiss LSM710 confocal microscope equipped with 488- and 561-nm lasers. Cells were kept at 37 °C during imaging using a heated stage. Membrane translocation in live cells was elicited by the addition of 400–500 nm PMA (Sigma), which was freshly prepared as a 4 μm PMA stock in HBSS from a 1 mm PMA stock solution in DMSO. Membrane translocation was monitored by imaging cells every 20–30 s, and the cytosolic fluorescence signal was quantified by integration in ImageJ. Depletion of cytosolic fluorescence was fitted to a logistic fit function to allow determination of the half-maximum translocation time, *t*_½_. The effects of the mutations were judged by the ratio of *t*_½_ between WT and mutant/truncation constructs.

For determination of *t*_½_, raw intensities were fitted; for easier visual comparison of the translocation curves, the individual data points and fit functions were normalized to the maximum and minimum cytoplasmic fluorescence signal.

### Radiometric kinase assay

The protein kinase A assay was carried out similarly to what has been described previously (48). Briefly, reactions were set up in 20 mm Tris, pH 7.5, 150 mm KCl, 10 mm MgCl_2_, 1% glycerol, 1 mm TCEP with 100 nm PKD1^CAT^ and varying substrate concentrations (syntide 2, obtained from Sigma, or the peptide corresponding to the PKD1 activation loop sequence (EKSFRRSVVGTPAYL), obtained from GenScript). Upon the addition of 1 mm ATP, added to [γ-^32^P]ATP, the reaction was started and carried out for 10 min at 30 °C. Each reaction was then spotted onto a 2 × 2-cm piece of nitrocellulose. The nitrocellulose snippets were washed three times with 75 mm phosphoric acid and transferred to scintillation counter tubes filled with 4 ml of Milli-Q water. The incorporation of γ-^32^P was read out by Cherenkov counting on a TRI CARB 21000 TR liquid scintillation counter. The counts were corrected by subtracting the background signal (reactions without substrate) and plotted against the substrate concentration. Data points were fitted to a Michaelis–Menten kinetic model, if feasible.

## Author contributions

D. J. E. and T. A. L. conceived the study and designed all of the experiments. D. J. E. carried out most of the experiments. Purification of the PKD1 kinase domain and autophosphorylation were carried out by K. M. S. with support from D. J. E. and T. A. L. SAXS data collection and analysis as well as structure refinement were done by T. A. L. M. H. conceived the data collection strategy for phosphopeptide mapping of PKD1 and parallel reaction monitoring of PKD1. T. G. processed the samples and conducted the data acquisition, and M. H. and T. G. both analyzed the data. D. J. E. wrote the manuscript with help from T. A. L. All authors read and approved the final manuscript.

## Supplementary Material

Supporting Information

## Footnotes

3 The abbreviations used are:

PKD: protein kinase D
SEC: size-exclusion chromatography
MALS: multi-angle light scattering
TGN: *trans*-Golgi network
PH: pleckstrin homology
CAMK: Ca^2+^/calmodulin-dependent kinase
DAG: diacylglycerol
PLC: phospholipase C
GPCR: G protein–coupled receptor
ULD: ubiquitin-like domain
PDB: Protein Data Bank
RBD: Ras-binding domain
r.m.s.d.: root mean square deviation
PRM: parallel reaction monitoring
SAXS: small-angle X-ray scattering
HSQC: heteronuclear single quantum coherence spectroscopy
PDGF: platelet-derived growth factor
PMA: phorbol 12-myristate 13-acetate
PKC: protein kinase C
TCEP: tris(2-carboxyethyl)phosphine
TEV: tobacco etch virus
HBSS: Hanks' balanced salt solution
NTD: N-terminal domain
EGFP: enhanced GFP
Chk2: checkpoint kinase 2.
