# Supplementary material for: A ubiquitin-like domain controls protein kinase D dimerization and activation by trans-autophosphorylation
Source: J Biol Chem. 2019 Aug 12;294(39):14422–41. doi: 10.1074/jbc.RA119.008713 (PMC6768651; doi:10.1074/jbc.RA119.008713)
Supplement: Supporting Information [file supp_294_39_14422__index.html]

A ubiquitin-like dimerization domain controls protein kinase D dimerization and activation by trans-autophosphorylation — A ubiquitin-like domain controls protein kinase D activation — A ubiquitin-like domain controls protein kinase D dimerization and activation by trans-autophosphorylation — A ubiquitin-like domain controls protein kinase D activation — Supporting Information 

# A ubiquitin-like domain controls protein kinase D dimerization and activation by trans-autophosphorylation

## Supporting Information

- Supporting Information (to be published online) - Phosphopeptide mapping of autophosphorylated PKD1 kinase domain.
- Supporting Information (to be published online) - Phosphopeptide mapping of autophosphorylated PKD1 kinase domain.
- Supporting Information (to be published online) - Parallel reaction monitoring mass spectrometry of PKD1 in mammalian cells.
- Supporting Information (to be published online) - Supporting Information file containing Figures S1-S5, Tables S1-S2, and Supporting Experimental Procedures.
