# Supplementary material for: A ubiquitin-like domain controls protein kinase D dimerization and activation by trans-autophosphorylation
Source: J Biol Chem. 2019 Aug 12;294(39):14422–41. doi: 10.1074/jbc.RA119.008713 (PMC6768651; doi:10.1074/jbc.RA119.008713)
Supplement: Supporting Information [file supp_RA119.008713_144682_2_supp_373491_pvrp3b.pdf]

A ubiquitin-like domain controls protein kinase D activation by trans-autophosphorylation

Daniel J. Elsner, Katharina M. Siess, Thomas Gossenreiter, Markus Hartl, Thomas A. Leonard

Supporting information

Material included:

Figures S1-S5

S1. The PKD N-terminus is conserved among metazoans.

S2. The ULD-C1a domain adopts a conventional β-grasp fold.

S3. The ULD forms concentration-dependent dimers.

S4. Electron density maps of the ULD-C1a interface.

S5. The PKD1^{CAT} autophosphorylation site can be mapped to S^{742}.

Tables S1-S2

Table S1. Data collection and refinement statistics

Table S2. Small-angle X-ray scattering sample information

Extended Experimental Procedures

Extended Experimental Procedures References

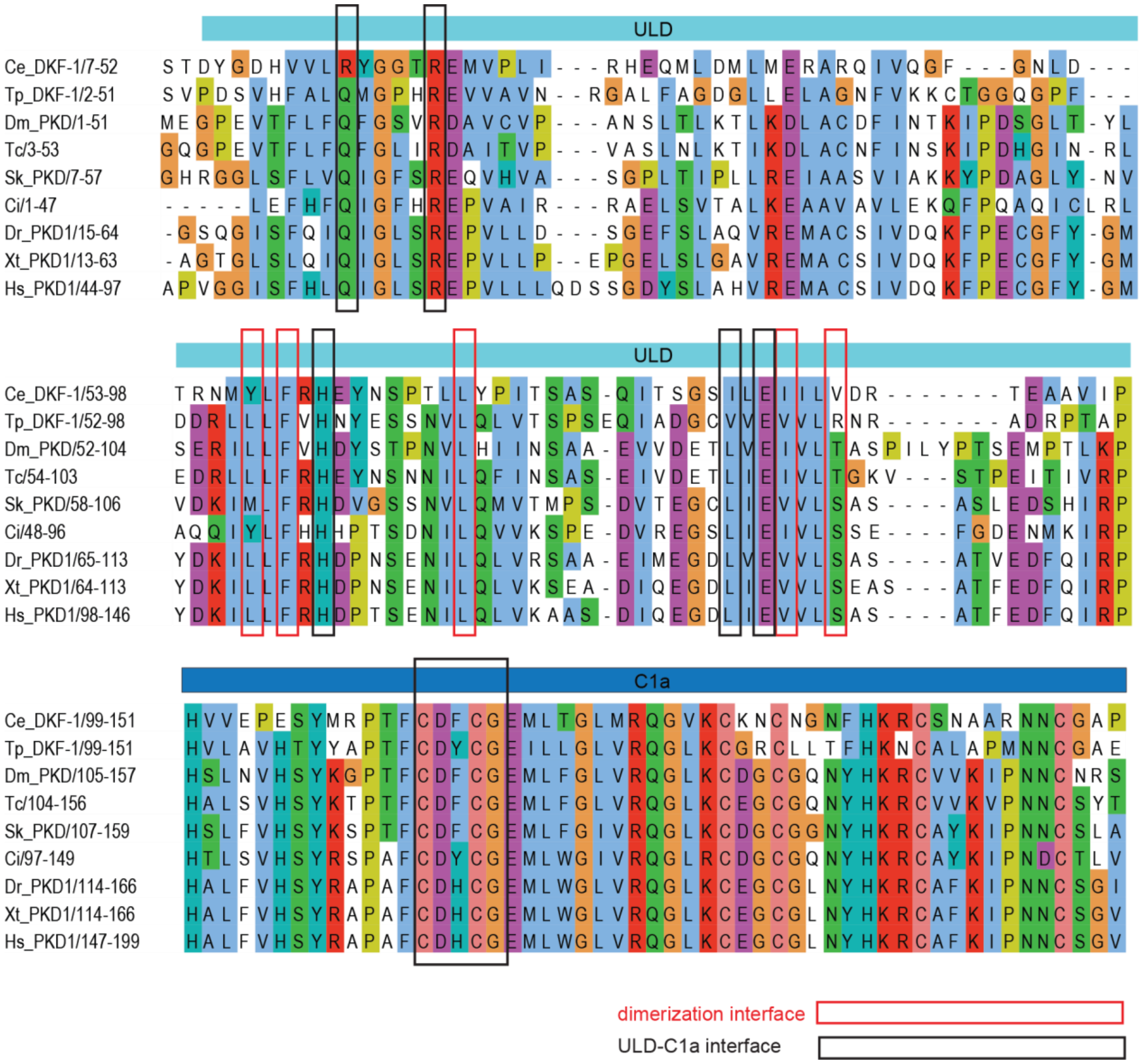

Figure S1. The PKD N-terminus is conserved among metazoans.

Alignment showing PKD isoforms from distantly related animal species: Ce: Caenorhabditis elegans, Tp: Trichinella patagoniensis, Dm: Drosophila melanogaster, Tc: Tribolium castaneum, Sk: Saccoglossus kowalevskii, Ci: Cionia intestinalis, Dr: Danio rerio, Xt: Xenopus tropicalis, Hs: Homo sapiens. Amino acids in the dimerization interface are highlighted with red boxes, amino acids at the ULD-C1a interface with black boxes.

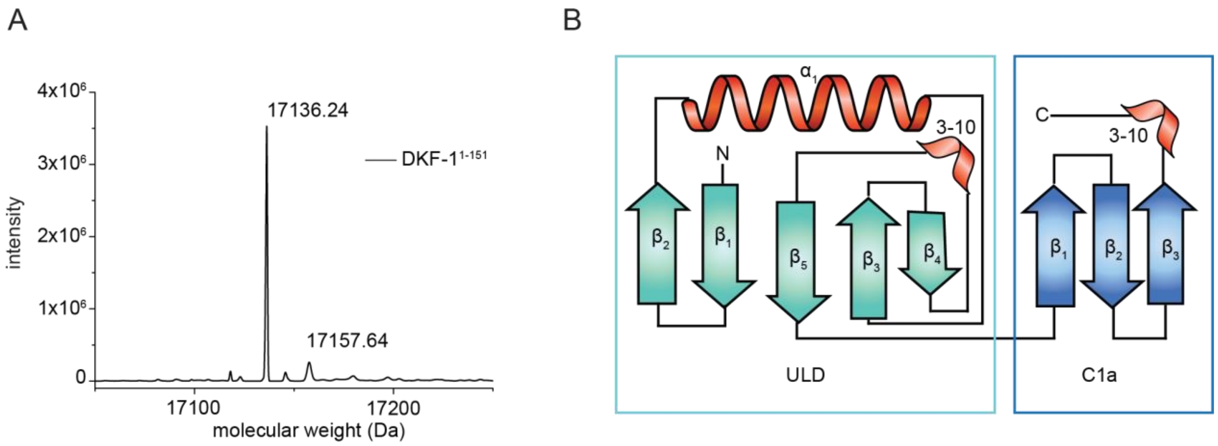

Figure S2. The ULD-C1a domain adopts a conventional β-grasp fold.

A. Mass spectrometry of the crystallized protein (DKF-$1^{1\text{-}151}$). The determined mass of 17136.24 Da, closely matches the expected mass of 17136.45 (-0.21 Da).

B. Secondary structure representation of the ULD-C1a crystal structure. The ULD adopts a typical β-grasp fold containing five antiparallel β-sheets (β_1- β_5) and one alpha-helix (α_1). The C1a domain contains 3 antiparallel β-sheets (β_1- β_3). Both domains also contain a short 3-10-helix element.

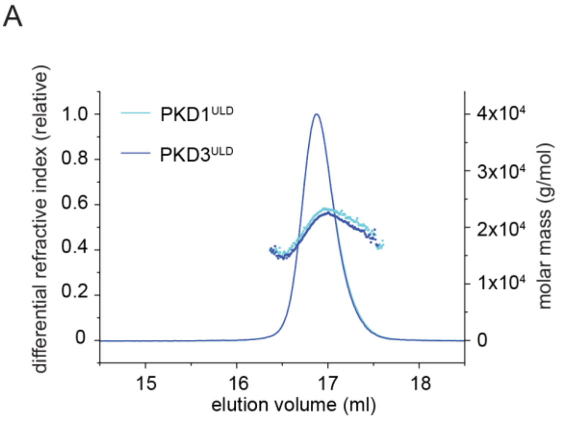

	poly-dispersity	$Mr_{exp.}$ (kDa)	$Mr_{theor.}$ (kDa)	$Mr_{exp.}$ / $Mr_{theor.}$
PKD1ULD	1.012	21.09	10.97	1.92
PKD3ULD	1.013	20.21	10.79	1.87

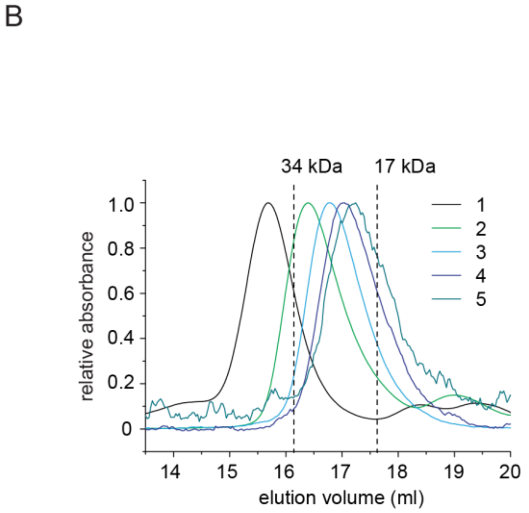

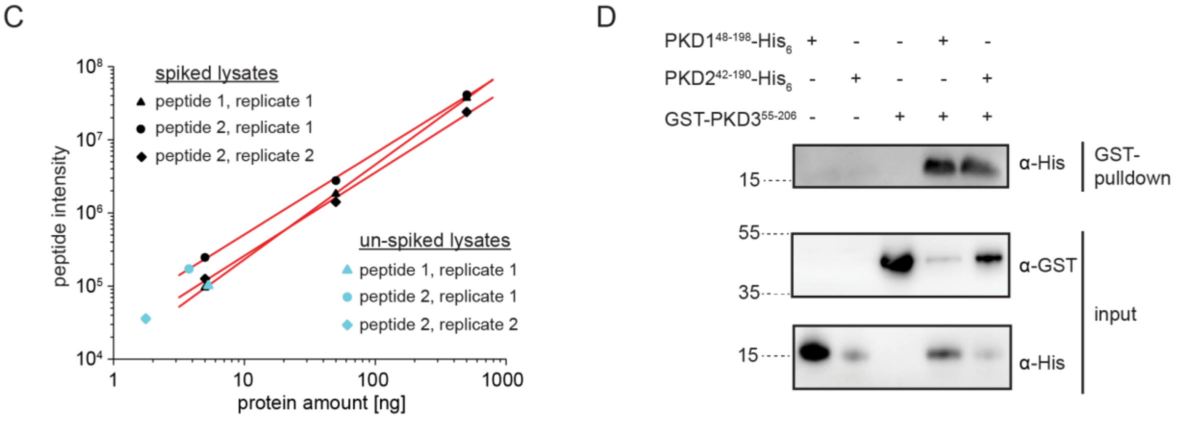

Figure S3. The ULD forms concentration-dependent dimers.

A. Static light scattering (SEC-MALS) of the human PKD1 ULD (PKD1^{ULD}, light blue) and PKD3 ULD (PKD3^{ULD}, dark blue) and their corresponding molar mass (dotted). The table summarizes the polydispersity (Mn/Mw) as well as the experimentally determined molecular weight (M_{exp}), the theoretical monomeric molecular weight (M_{theo}) and the oligomeric state (M_{exp}/M_{the}) of both proteins (see as well Fig 2B).

B. Overlay of elution profiles of recombinant unlabelled or FITC-labelled DKF-1 ULD-C1a from a S200 10/300 size exclusion column. Serial runs were made by reinjecting the peak fraction. Elution of unlabelled protein was monitored by absorbance at 280 nm, labelled protein at 485 nm. 100 µM unlabelled ULD-C1a (1), 40 µM FITC-labelled ULD-C1a (2), 4 µM FITC-labelled ULD-C1a (3), peak of elution 3 (4), peak of elution 4 (5). Dashed lines indicate the expected elution volumes for a globular, monomeric (17 kDa) or dimeric (34 kDa) protein of the same molecular weight.

C. Intensities of identical peptides between rat and human PKD1 in INS-1 lysates targeted by parallel reaction monitoring mass spectrometry. Lysates were spiked with an equimolar amount (5, 50, 500 ng) of human PKD1$^{ULD-C1a}$ and PKD1^{PH-CAT} (black symbols) or un-spiked (cyan symbols). The intensity of the un-spiked lysates was subtracted from the spiked samples prior to regression analysis. The linear regressions (red, solid line) were used to calculate the amounts of endogenous PKD1 protein. The experiment was repeated twice (replicate 1/replicate 2). Note that one of the peptides (peptide 1) could only be reliably detected in all samples in one replicate, and that some of the endogenous peptides are below the lowest amount of spiked protein and therefore cannot be reliably quantified.

D. PKD1, PKD2 and PKD3 heterodimerize via their ULD domains. ULD-C1a homologs of human PKD1, PKD2, and PKD3 were expressed in bacteria either as C-terminal His_6 ($PKD1^{48\text{-}198}$, $PKD2^{42\text{-}190}$) or as N-terminal GST fusion ($PKD3^{55\text{-}206}$). All proteins were expressed individually and His_6-tagged and GST-tagged proteins were co-expressed. GST tagged proteins were pulled down from the lysates using glutathione beads and probed for His_6-tagged proteins by immunoblot.

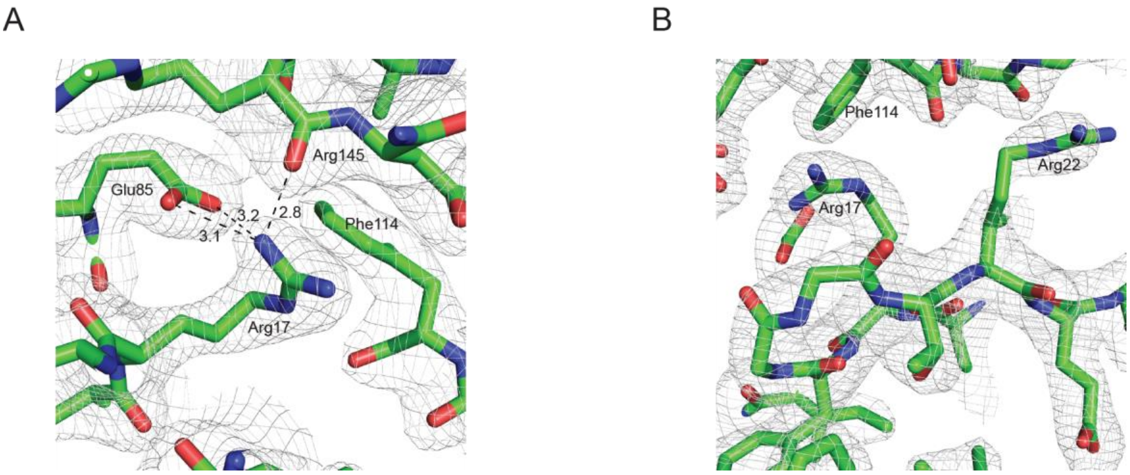

Figure S4. Electron density maps of the ULD-C1a interface.

A. 2F_o-F_c electron density map surrounding Arg17 of the ULD. Arg17 makes a bifurcated hydrogen bond to the side chain carboxylate of Glu85 (ULD) and a hydrogen bond to the backbone carbonyl of Arg145 (C1a). Clear electron density is observed for Phe114 of the C1a domain, which forms a hydrogen bond between its backbone carbonyl and the epsilon nitrogen of Arg17.

B. 2F_o-F_c electron density map surrounding both Arg17 and Arg22 of the ULD. In contrast to Arg17, the electron density for Arg22 is less well defined, though a clear region of electron density could be observed at a location, size, and shape consistent with the guanidino group of the side chain. Contouring of the 2F_o-F_c map at 0.7σ reveals contiguous electron density for the side chain.

A

ArgC digest - sequence coverage (61.3%)

```
GARQIQENVD ISTVYQIFPD EVLGSGQFGI
VYGGKHRKTG RDVAIKIIDK LRFPTKQESQ
LRNEVAILQN LHHPGVVNLE CMFETPERVF
VVMEKLHGDM LEMILSSEKG Rlpehitkfl
itqilvalrh lhfknivhcd lkpenvllas
adpfpqvklc dfgfarIIGE KSFRRSVVGT
PAYLAPEVLR nkgynrsldm wsvgviiyvs
lsgtfpfned edihdqiqna afmyppnpwk
eisheaidli nnllqvkmrk rYSVDKTLSH
PWLQDYQTWL DLRELECKIG ERYITHESDD
LRWEKYAGEQ RLQYPTHLIN PSASHS
```

phosphopeptide mapping

phospho site	phosphopeptide identified	local. prob.	score	mass error (ppm)	intensity (x10⁶)	ratio mod/base
**S742**	pSVVGTPALAPEVLR	1.00	221.9	-0.31	11844	0.408
**S738**	IIGEKpSFRR	1.00	215.1	-0.32	1169	0.121
S890	LQYPTHLINPSApSHS	1.00	267.6	0.28	735	0.015
Y749	SVVGTPApYLAPEVLR	1.00	222.9	-0.20	776	0.027
S892	LQVPTHLINPSASHpS	1.00	311.4	0.28	447	0.009
T746	SVVGpTPAYLAPEVLR	1.00	181.3	-0.20	62	0.002
S888	LQYPTHLINPpSASHS	1.00	250.3	-0.04	39	<0.001
S864	YITHEpSDDLRWEKYAGEQR	1.00	189.3	0.25	13	<0.001
S835	YSVDKTLpSHPWLQDYQTWLDLR	1.00	271.1	0.17	5	0.023
S625	FPpTKQESQLR	0.56	94.5	0.99	1	<0.001
S880	LQpYPTHLINPSASHS	0.95	96.0	4.29	1	<0.001

B

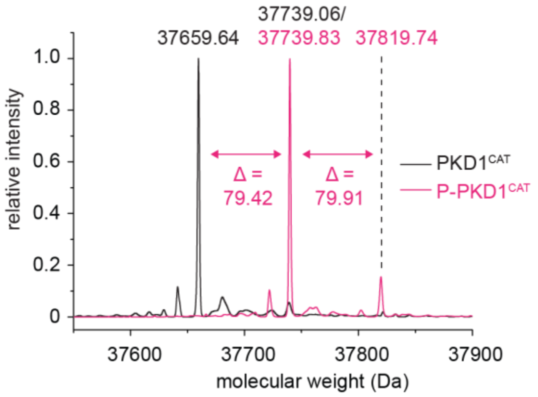

ArgC digest - sequence coverage (60.4%)

```
garQIQENVD ISTVYQIFPD EVLGSGQFGI VYGGKHRktg
rDVAIKIIDK lrFPTKQESQ LRNEVAILQN LHHPGVVNLE
CMFETPERVF VVMEKLHGDM LEMILSSEKG RLPEHITKFL
ITQILVALRh lhfkNIVHCD LKPENVLLAS ADPFPQVKLC
DFGFARIIGE KSFRRSVVGT PAYLAPEVLR nkgynrsldm
wsvgviiyvs lsgtfpfned edihdqiqna afmyppnpwk
eisheaidli nnllqvkmrk rYSVDKTLSH PWLQDYQTWL
DLRELECKIG ERYITHESDD LRWEKYAGEQ RLQYPTHLIN
PSASHS
```

phosphopeptide mapping

phospho site	phosphopeptide identified	local. prob.	score	mass error (ppm)	intensity (x10⁶)	ratio mod/base
**S742**	pSVVGTPALAPEVLR	1.00	242.8	-1.01	3099	13.881
**S738**	IIGEKpSFRR	1.00	173.3	0.22	1005	0.974
S888	LQYPTHLINPpSASHS	0.99	140.8	0.30	7.7	<0.001
S890	LQYPTHLINPSApSHS	0.84	138.5	0.02	1	<0.001

Figure S5. The PKD1^{CAT} autophosphorylation site can be mapped to S^{742}.

A. ArgC digest of ATP incubated protein (see Figure 7D) analysed by LC-MS/MS. Identified peptides mapped onto the protein sequence shown in magenta. The table lists all PKD-specific phospho-sites identified with assigned localization probability, score and mass error (in parts per million), in order of signal intensity. The phosphorylation site within the respective peptide is highlighted in magenta. Score refers to the Andromeda (Max Quant) score of the best scoring peptide spectrum match assigned to each site. For each phospho-site the ratio of phosphorylated peptide to unmodified peptide signal (ratio mod/base) is reported.

B. Overlay of intact mass spectra of recombinant PKD1^{CAT} and stoichiometrically phosphorylated PKD1^{CAT} (P-PKD1^{CAT}) which was generated as described in the methods. The shift in the molecular mass of about 80 Da indicates the protein is predominantly monophosphorylated, and to a smaller extent also bisphosphorylated. LC-MS/MS analysis of ArgC digested protein as described in Fig S3A, showing that monophosphorylation for PKD1^{CAT} is primarily located on S^{742}.

Table S1. Data collection and refinement statistics

Data collection	*Native*	*Zinc SAD*
space group	$P2_12_12$	$P2_12_12$
unit cell (*a*, *b*, *c* in Å)	45.41, 83.02, 37.08	45.55, 82.21, 37.06
wavelength (Å)	0.97626	1.28254
resolution range (Å)	50 – 2.3	45 – 2.9
observations	44,855	17,344
unique reflections	6,939	3,347
completeness (%)[a]	99.4 (97.0)	98.6 (95.8)
multiplicity	6.3 (5.9)	5.2 (4.7)
I/σ_I	11.2 (1.6)	10.9 (1.7)
R_{pim} (%)	3.9 (53.0)	4.5 (48.4)
$CC_{1/2}$	0.997 (0.619)	0.998 (0.847)

Phasing statistics

No. Zn sites finally modeled	2
Site occupancies	1.000, 0.911
Figure of merit (FOM)	0.33

Structure refinement

resolution range	57.1 – 2.3
reflections used	6,913
R-factor, R_{free}[b] (%)	23.2 (27.6)
modelled residues	12-149
rms deviations	
bond lengths (Å)	0.009
bond angles (deg)	1.071

Ramachandran

Residues in favored region	97.1%
Residues in outlier region	0.74%

[a] Values in parentheses refer to the highest resolution shell.
[b] R_{free} = free *R*-factor based on random 5% of all data.

Table S2. Small-angle X-ray scattering sample information

DKF-1 ULD-C1a	*Information*
Source	Recombinant expression in *E.coli*
Purification	GST-affinity chromatography, Superdex 75 size-exclusion chromatography
Composition of sample	UNIPROT Q9XUJ7-1 residues 1-151<br>17.14 kDa
Buffer composition	20 mM Tris, pH 8.0<br>100 mM NaCl<br>1 mM TCEP
Sample concentration	6.5 mg/ml (380 µM, concentration of monomers)<br>ε = 8 940 M^{-1} cm^{-1} (λ = 280 nm)
SEC-SAS	In-line SEC-SAS, BM29 ESRF<br>Superdex 200 10/300<br>Injection: 250 µl of 6.5 mg/ml<br>Flow rate: 0.5 ml min^{-1}
SAS-independent measure of dispersity	SEC-MALS<br>Polydispersity = 1.004<br>Mr = 33.34 kDa<br>$Mr_{exp.}/Mr_{theor.}$ = 1.95 (dimer)

Extended Experimental Procedures

Peptide mass spectrometry and phosphorylation site mapping

1 µg of protein was denatured in 8 M urea 50 mM ammonium bicarbonate (ABC), reduced with 10 mM DTT for 15 min at room temperature, alkylated with 20 mM IAA for 20 min at room temperature in the dark, excessive IAA quenched with 10 mM DTT and then diluted to 0.6 M urea with 50 mM ABC. After addition of 10 mM $CaCl_2$ the protein was digested overnight using mass-spec grade ArgC (Roche) at 37°C. The digestion was stopped with 1% trifluoroacetic acid (TFA) and the peptides were desalted using custom-made C18 stagetips (1). The peptides were separated on an Ultimate 3000 RSLC nano-flow chromatography system (Thermo-Fisher), using a pre-column for sample loading (PepMapAcclaim C18, 2 cm × 0.1 mm, 5 µm, Dionex-Thermo-Fisher), and a C18 analytical column (PepMapAcclaim C18, 50 cm × 0.75 mm, 2 µm, Dionex-Thermo-Fisher), applying a linear gradient from 2% to 30% solvent B (80% acetonitrile, 0.1% formic acid; solvent A 0.1% formic acid) at a flow rate of 230 nl/min over 60 min. Eluting peptides were analysed on a Q Exactive HF Orbitrap mass spectrometer (Thermo Fisher), equipped with a Proxeon nano-spray-source (Thermo Fisher), operated in data-dependent mode. Survey scans were obtained in a mass range of 380-1650 m/z with lock mass on, at a resolution of 120.000 at 200 m/z and an AGC target value of 3E6. The 10 most intense ions were selected with an isolation width of 2 Da, fragmented in the HCD cell at 27% collision energy and the spectra recorded at a target value of 1E5 and a resolution of 30000. Peptides with a charge of +1 were excluded from fragmentation, the peptide match option was set to “preferred”, the exclude isotope feature was enabled, and selected precursors were dynamically excluded from repeated sampling for 15 seconds. The phosphorylation site mapping was performed once for each protein preparation.

Raw data were processed using the MaxQuant software package (version 1.6.0.16, http://www.maxquant.org/) (2) and searched against a custom database containing the PKD1^{CAT} (569-892) target sequence, the Bombyx mori reference proteome (UniProt), Spodoptera proteins (UniProt), and common contaminants (containing 28512, 14785, and 379 entries, respectively). The search was performed with full ArgC specificity and a maximum of two missed cleavages. Oxidation of methionine, N-terminal acetylation, carbamidomethylation of cysteine and phosphorylation of serine, threonine and tyrosine were defined as variable modifications – all other parameters were set to default. Search results were filtered at 1% FDR on PSM (peptide spectrum match), peptide and protein level. All top-scoring spectra of PKD1 phosphopeptides were manually validated.

Quantification of PKD1 using parallel reaction monitoring

HEK293T and INS-1 cells were trypsinized and resuspended in growth medium. Cells were counted and aliquoted to 1.5-2.0 *10^6 cells (HEK293T) or 3.26-3.79 *10^6 cells (INS-1). Cells were lysed in RIPA buffer (10 mM Tris pH 7.5, 225 mM NaCl, 0.1% SDS, 1.0% Triton X-100, 0.5% sodium deoxycholate, 1x protease inhibitor cocktail (Roche), 1 mM TCEP) and frozen in liquid nitrogen. Cell lysates were thawed on ice and insoluble material was spun down by centrifugation with 16 000 g at 4°C for 30 min. To generate an external three-point calibration curve samples were spiked with increasing amounts (5, 50, 500 ng/lysate) of recombinant PKD1 protein, which consists of an equimolar mixture of the two recombinant proteins PKD1$^{ULD\text{-}C1a}$ and PKD1$^{PH\text{-}CAT}$. Proteins were then precipitated by addition of 500 µl ice cold acetone and incubated for at least 15 min at -20°C. Precipitated protein was spun down (500 g, 4°C, 30 min) and washed with ice cold 80% acetone

(500 µl). After another centrifugation step (500 g, 4°C, 30 min) acetone was removed completely and the protein pellet was air-dried. The protein pellet was denatured in 8 M urea 50 mM ABC to a protein concentration of 2 µg/µl, reduced with 10 mM DTT for 30 min at room temperature, alkylated with 20 mM IAA for 30 min at room temperature in the dark, quenched with 5 mM DTT, diluted to 4 M urea with 50 mM ABC, digested with LysC protease for 3 hours, diluted to 1 M urea with 50 mM ABC and digested with trypsin overnight at 37°C. The digestion was stopped with 1% TFA and the peptides were desalted using custom-made C18 stagetips (1).

The parallel reaction monitoring (PRM) assay was set-up based on shotgun measurements of the recombinant PKD1 spike-in, selecting initially eight high intensity proteotypic peptides in a single charge state, with no missed cleavages, no methionine, and an even distribution over the chromatographic gradient. PRM assay generation was performed using Skyline (3), resulting in a scheduled assay with 6-8 min windows. For data acquisition we operated the same liquid-chromatography system under the same conditions as for the peptide mapping but coupled to an Orbitrap Fusion Lumos instrument via a customised EASY-spray source (both Thermo Fisher) with coated silica emitters (New Objective). MS parameters: survey scan with 15k resolution, AGC 2E5, 50 ms IT, over a range of 380 to 1400 m/z; PRM scan with 60k resolution, AGC 2E5, 700 ms IT, isolation window of 0.7 m/z with 0.2 m/z offset, and NCE of 32%.

The spectral library for PRM data analysis in Skyline was generated as follows: peptides from one replicate with the highest spike-in concentration were identified using X!Tandem version Vengeance (2015.12.15.2) (4). The search was conducted using SearchGUI version 3.3.5 (5) against the PKD1 target sequence alone, as a target/decoy approach is not applicable to PRM data. The identification settings were as follows: Trypsin, specific, with a maximum of 2 missed cleavages; 10.0 ppm as MS1 and 20.0 ppm as MS2 tolerances; Carbamidomethylation of cysteine was set as a fixed and acetylation of protein N-term and oxidation of methionine as variable modifications. Peptides and proteins were inferred from the spectrum identification results using PeptideShaker version 1.16.31 (6). Given the small database size, FDR validation was omitted and instead the PKD1 spectra manually inspected in PeptideShaker, exported as mzident.ml files and imported to Skyline using a score cut-off of 0.95. The spectra matching transitions at the peak apex were further manually validated in Skyline, with all peptides showing plausible fragmentation spectra, consistent with the shotgun measurements and concentration-dependent behaviour in the dilution series.

Data analysis, including manual validation of all peptides and their transitions (based on retention time, relative ion intensities, and mass accuracy), and relative quantification was performed in Skyline. Three of the eight target peptides were removed from the analysis due to strong interfering signals from the background matrix at lower concentrations, or in case there were less than three transitions detectable over the whole concentration range. Up to ten of the most intense non-interfering transitions of the five target peptides were selected and their peak areas were summed for peptide quantification (total peak area). Peptide intensities were summed to protein intensities. To correct for minor variations in sample injection amounts and instrument stability, the total ion current of all MS1 scans between 10 and 60 min of the gradient was summed for each run and used as a global normalization factor. The signals of un-spiked samples were subtracted from those of the dilution series of spiked samples and scaled for easier interpretation. The decimal logarithm of the signal intensity was plotted against the decimal logarithm of the spiked protein concentration and Origin 7 was used to fit the double log-plot data points to a linear regression model with the formula $y = A + B*x$ (Fig 2D, Fig S2D). The linear regression was used to calculate the amount of endogenous protein per cell. The molar concentration of cellular PKD1 was

then calculated assuming a total cell volume of 1800 μm^3 for HEK293T cells (7). Since a large proportion of these cells can be assigned to the nucleus, a substantially smaller volume of 822 μm^3 was assumed for the cytosolic volume of these cells (7).

As a rough assessment of the cellular concentration range of PKD1 in INS-1 cells the PRM assay was repeated in two biological replicates based upon the three peptide sequences shared between rat and human. Of these three peptides two could be used for quantification according to the same criteria as described above, although only one peptide could be detected in all spiked and un-spiked samples in both biological replicates. Therefore, peptide intensities were not summed but analysed individually and the data was not globally normalized. The un-spiked peptide signals were subtracted from the spiked samples before performing linear regression analysis. From the regression the amount of endogenous protein per lysate was calculated. The cellular molar concentration of PKD1 in INS-1 cells was calculated using the molecular weight of the spiked proteins, the number of cells that had been counted prior to lysis and the volume of a rat beta cell of 1020 μm^3 which has been previously reported (8).

Experimental design and statistical rational of mass spectrometry experiments

The applied PRM analysis falls in the Tier 3 category (9). Lysates and external reference curve standards were prepared and measured in three biological replicates for HEK293T cells, which should allow a reasonable estimation of the concentration range. The additional experiment on INS-1 cells should serve only as a rough estimate of the concentration range without offering the full quantitative accuracy of the HEK293T experiment. The non-spike-in lysate samples were run before the spike-in standards to prevent carry-over and blank runs were used to determine baseline signal. The standards were measured in the order of increasing concentration. Injection of 25 ng of HeLa digest on a 30 min gradient were used to monitor system performance before and after the sample batch by determining number of PSMs and other performance parameters as described in (10) showing no significant differences over the course of the experiment. In addition, all samples were globally normalized to total ion current as described above.

The mass spectrometry proteomics data have been deposited to the ProteomeXchange Consortium (http://proteomecentral.proteomexchange.org) via the PRIDE partner repository (11) with the dataset identifier PXD013216 and PXD013232.

Extended Experimental Procedures References

1. Rappsilber, J., Mann, M., and Ishihama, Y. (2007) Protocol for micro-purification, enrichment, pre-fractionation and storage of peptides for proteomics using StageTips. *Nat. Protoc.* **2**, 1896–1906

2. Cox, J., and Mann, M. (2008) MaxQuant enables high peptide identification rates, individualized p.p.b.-range mass accuracies and proteome-wide protein quantification. *Nat. Biotechnol.* **26**, 1367–1372

3. MacLean, B., Tomazela, D. M., Shulman, N., Chambers, M., Finney, G. L., Frewen, B., Kern, R., MacCoss, M. J., Tabb, D. L., Liebler, D. C., and MacCoss, M. J. (2010) Skyline: an open source document editor for creating and analyzing targeted proteomics experiments. *Bioinformatics*. **26**, 966–968

4. Craig, R., and Beavis, R. C. (2004) TANDEM: Matching proteins with tandem mass spectra. *Bioinformatics*. **20**, 1466–1467

5. Vaudel, M., Barsnes, H., Berven, F. S., Sickmann, A., and Martens, L. (2011) SearchGUI: An open-source graphical user interface for simultaneous OMSSA and X!Tandem searches. *Proteomics*. **11**, 996–999

6. Burkhart, J. M., Oveland, E., Sickmann, A., Berven, F. S., Barsnes, H., Vaudel, M., Martens, L., and Zahedi, R. P. (2015) PeptideShaker enables reanalysis of MS-derived proteomics data sets. *Nat. Biotechnol.* **33**, 22–24

7. Bruggeman, F. J., Schwabe, A., Verschure, P. J., Crémazy, F., and Kempe, H. (2014) The volumes and transcript counts of single cells reveal concentration homeostasis and capture biological noise. *Mol. Biol. Cell*. **26**, 797–804

8. Finegood, D. T., Scaglia, L., and Bonner-Weir, S. (1995) Dynamics of β-cell mass in the growing rat pancreas: Estimation with a simple mathematical model. *Diabetes*. **44**, 249–256

9. Carr, S. A., Bethem, R., Neubert, H., Deutsch, E. W., Ackermann, B. L., Kuhn, E., Rifai, N., Botelho, J., Paulovich, A. G., Abbatiello, S. E., Aebersold, R., Liu, T., Moritz, R., Blonder, J., Van Eyk, J., Srinivas, P. R., Koomen, J. M., Domon, B., Kinsinger, C., Mani, D., Mansfield, E., Borchers, C., Ritchie, J., Boja, E., MacLean, B., Townsend, R. R., Anderson, L., Lee, J. S. H., Reiter, L., Bradshaw, R. A., Weintraub, S., Burlingame, A. L., Rodriguez, H., Boyne, M., Oses-Prieto, J., Hüttenhain, R., Hoofnagle, A. N., Grant, R. P., Chan, D., Whiteley, G., Keshishian, H., Lee, S.-W., Wiita, A., Vitek, O., and Liebler, D. C. (2014) Targeted Peptide Measurements in Biology and Medicine: Best Practices for Mass Spectrometry-based Assay Development Using a Fit-for-Purpose Approach. *Mol. Cell. Proteomics*. **13**, 907–917

10. Weilnböck, L., Luider, T. M., Huber, C. G., Dusberger, F., Stingl, C., Mazanek, M., Köcher, T., Straube, W. L., Pichler, P., and Mechtler, K. (2012) SIMPATIQCO: A Server-Based Software Suite Which Facilitates Monitoring the Time Course of LC–MS Performance Metrics on Orbitrap Instruments. *J. Proteome Res.* **11**, 5540–5547

11. Xu, Q.-W., Csordas, A., Mayer, G., Griss, J., Lavidas, I., Ternent, T., Dianes, J. A., Vizcaíno, J. A., Perez-Riverol, Y., Reisinger, F., Hermjakob, H., Wang, R., and del-Toro, N. (2015) 2016 update of the PRIDE database and its related tools. *Nucleic Acids Res.* **44**, D447–D456
